# Genetic Analysis in *Drosophila* Reveals a Role for the Mitochondrial Protein P32 in Synaptic Transmission

**DOI:** 10.1534/g3.111.001586

**Published:** 2012-01-01

**Authors:** Andrew Lutas, Christopher J. Wahlmark, Shaona Acharjee, Fumiko Kawasaki

**Affiliations:** * Department of Biology; † Center for Molecular Investigation of Neurological Disorders, Pennsylvania State University, University Park, Pennsylvania 16802

**Keywords:** calcium, neurotransmitter release, neuromuscular, temperature sensitive, dorsal longitudinal flight muscle

## Abstract

Mitochondria located within neuronal presynaptic terminals have been shown to play important roles in the release of chemical neurotransmitters. In the present study, a genetic screen for synaptic transmission mutants of *Drosophila* has identified the first mutation in a *Drosophila* homolog of the mitochondrial protein P32. Although P32 is highly conserved and has been studied extensively, its physiological role in mitochondria remains unknown and it has not previously been implicated in neural function. The *Drosophila* P32 mutant, referred to as *dp32^EC1^*, exhibited a temperature-sensitive (TS) paralytic behavioral phenotype. Moreover, electrophysiological analysis at adult neuromuscular synapses revealed a TS reduction in the amplitude of excitatory postsynaptic currents (EPSC) and indicated that dP32 functions in neurotransmitter release. These studies are the first to address P32 function in *Drosophila* and expand our knowledge of mitochondrial proteins contributing to synaptic transmission.

Previous studies have established important functions for mitochondria in synaptic transmission. A key aspect of this work has focused on the roles of mitochondria in providing energy for synaptic function [reviewed in Kann and Kovács (2007) and Mattson *et al.* (2008)]. Moreover, mitochondria are known to sequester and release calcium, and earlier studies have addressed roles for these mechanisms in presynaptic calcium signaling and neurotransmitter release (Billups and Forsythe 2002; Chouhan *et al.* 2010; David and Barrett 2003; Klose *et al.* 2009; Tang and Zucker 1997; Yang *et al.* 2003). This progress, along with recent work implicating mitochondrial mechanisms in neurological disease [reviewed in Mattson *et al.* (2008), Rezin *et al.* (2009), Schon and Przedborski (2011), Vos *et al.* (2010), and Whitworth and Pallanck (2009)], highlights the importance of understanding the molecular basis of mitochondrial function in synaptic transmission. The present study advances our understanding through identification of the first P32 mutant in a multicellular organism and by revealing a novel role for this mitochondrial protein in neurotransmitter release.

P32 was first identified in HeLa cells as a protein associated with the splicing factor ASF/SF2 (Krainer *et al.* 1991), and it was found to be conserved among eukaryotic organisms (Muta *et al.* 1997). Although many studies employing immunocytochemical and subcellular fractionation analysis have shown that P32 is primarily localized in the mitochondrial matrix (Dedio *et al.* 1998; Muta *et al.* 1997; Seytter *et al.* 1998), its expression in other compartments, including the extracellular cell surface, has been reported (Fogal *et al.* 2008; Ghebrehiwet *et al.* 2001; Majumdar *et al.* 2002; Matthews and Russell 1998). The crystal structure of P32 has provided valuable information (Jiang *et al.* 1999). Three P32 molecules form a doughnut-shaped homotrimeric structure that displays an unusually asymmetric charge distribution such that one surface of the doughnut is covered with negatively charged residues. On the basis of this structural information, the authors proposed that P32 may serve as a high-capacity divalent cation-binding protein within the mitochondrial matrix, may associate with the inner mitochondrial membrane in the presence of divalent metal ions or even regulate pore opening of channels in the inner mitochondrial membrane.

Previous studies have suggested P32 may function in a wide variety of processes, including RNA splicing (Krainer *et al.* 1991), complement system signaling [reviewed in Ghebrehiwet *et al.* (2001)], cell polarity determination (Bialucha *et al.* 2007), mitochondrial oxidative phosphorylation (Fogal *et al.* 2010; Muta *et al.* 1997) and apoptosis (Itahana and Zhang 2008; Rizvi *et al.* 2011; Sunayama *et al.* 2004). In addition, several studies have reported binding interactions of P32 with viral proteins (Beatch and Hobman 2000; Bruni and Roizman 1996; Luo *et al.* 1994; Matthews and Russell 1998; Wang *et al.* 1997; Yu *et al.* 1995). The first genetic analysis of P32 was carried out in yeast and led to contradictory conclusions with regard to P32 function in regulating oxidative phosphorylation (Muta *et al.* 1997; Seytter *et al.* 1998); thus, the physiological role of P32 in mitochondria remains unclear. To date, a major limitation in studies of P32 function has been the lack of mutants in multicellular model organisms. The present study reports identification and characterization of a *Drosophila* P32 mutant and further defines the molecular basis of mitochondrial function in neurotransmitter release.

## Materials and Methods

### *Drosophila* strains

*cac^TS2^*, *Appl-GAL4*, and *elav-GAL4 Appl-GAL4* were from our laboratory stock collection. The *UAS-mito-GFP* and *UAS-mCD8-mRFP* transgenic lines were obtained from the Bloomington Stock Center. Deficiency lines *Df(2R)14F06W-06*, *Df(2R)14F06W-07*, *Df(2R)14F06W-08*, and *Df(2R)14F06W-09* were generously provided by William Gelbart (Harvard University). A third chromosome *20xUAS-GCaMP3* line was kindly provided by Loren Looger [(Janelia Farm Research Campus, Howard Hughes Medical Institute (HHMI)]. *UAS-YC3.6*, *UAS-mito-DsRed*, *UAS-dp32*, *UAS-dp32-EGFP*, and *UAS-mito-GCaMP3* transgenic lines were generated in the current study (see *Generation of Transgenic Lines*). Wild-type flies were Canton-S. Stocks and crosses were cultured on a conventional cornmeal-molasses-yeast medium at 20°.

### Mutagenesis and screening

To isolate new mutations that enhance or suppress the *cac^TS2^* paralytic phenotype, a genetic screen was carried out as summarized in Figure S1. Briefly, *cac^TS2^* males with an isogenized second chromosome were exposed for 24 hr to 25 mM ethyl methanesulfonate (EMS) (Dellinger *et al.* 2000). F3 flies homozygous for a mutagenized second chromosome in a *cac^TS2^* genetic background were screened for altered *cac^TS2^* behavior at 36° as described previously (Brooks *et al.* 2002; Kawasaki *et al.* 2011).

### Molecular characterization of the dp32 mutant

Sequence analysis of candidate genes was carried out essentially as described previously (Kawasaki *et al.* 2011). Briefly, genomic DNA was prepared from the *dp32* mutant or flies carrying the parent second chromosome used in the mutagenesis. This was used as template for PCR, and gel-purified PCR products were sequenced at the Penn State University Nucleic Acids Facility.

### Generation of transgenic lines

Transformation constructs for *UAS-YC3.6*, *UAS-mito-DsRed*, *UAS-dp32*, and *UAS-dp32-EGFP* transgenic lines were generated by inserting the respective open reading frame (ORF) for YC3.6 (Nagai *et al.* 2004), dP32 (cDNA accession number AY094810), or dP32 with EGFP fused to its C-terminus, into the *P* element transformation vector, pUAST (Brand and Perrimon 1993). For *UAS-mito-DsRed* and *UAS-mito-GCaMP3*, a mitochondrial targeting sequence was fused to the N-terminus of the *DsRed* (Baird *et al.* 2000) or *GCaMP3* (Tian *et al.* 2009) ORFs. For *UAS-mito-DsRed*, the mitochondrial targeting sequence corresponded to the mitochondrial targeting domain of the human COX8 protein (Rizzuto *et al.* 1995). For *UAS-mito-GCaMP3*, the mitochondrial targeting sequence corresponded to amino acids 1-71 of the dP32 N-terminus. Generation of transgenic lines was achieved as described previously (Kawasaki *et al.* 2004). A cDNA clone for *Drosophila* gene CG6459, which we renamed *dp32*, was obtained from the *Drosophila* Genomics Research Center (Clone ID: LD29590). Neural expression of UAS transgenes was achieved using the *Appl-GAL4* driver or an *elav-GAL4 Appl-GAL4* double driver chromosome in the case of experiments for rescue of the *dp32^EC1^* mutant behavioral phenotype.

### Generation of a polyclonal anti-dP32 antibody

A rabbit polyclonal antiserum, anti-dP32, was generated against a synthesized peptide corresponding to the C-terminus of the dP32 sequence (amino acids 246–263; Figure 3) as in our previous work (Zou *et al.* 2008). Through a commercial service (Pocono Rabbit Farm and Laboratory, Canadensis, PA), the peptide was synthesized with cysteine appended at the N-terminus and prepared for injection by conjugation with keyhole-limpet hemocyanin (KLH).

### Western analysis

Western analysis of head homogenates was performed using conventional methods as described previously (Kawasaki *et al.* 2004; Zou *et al.* 2008). Briefly, the equivalent of one fly head was loaded per lane on a 12% SDS-PAGE gel. The primary antibodies, a rabbit polyclonal anti-dP32 antibody and a monoclonal anti-GFP antibody (Clontech, Mountain View, CA), were used at a dilution of 1:1,000. Detection was performed with horseradish peroxidase (HRP)-conjugated secondary antibodies (Amersham Biosciences, Arlington Heights, IL) and enhanced chemiluminescence (ECL Plus Western Blotting Detection System; Amersham Biosciences). Tubulin served as loading control and was detected using a monoclonal anti-acetylated α-tubulin antibody (Sigma, St. Louis, MO) at a dilution of 1:2,000,000.

For developmental Western blots, *Drosophila* embryos were collected every 4 hr in large culture cages and allowed to develop to the appropriate stage. Whole-animal homogenates were generated from several developmental stages. The amount of homogenate loaded per lane was a follows: early or late embryos (11 embryos), third instar larvae (the equivalent of 0.1 bodies), late pupae (the equivalent of 0.09 bodies), and adults (the equivalent of 0.1 bodies). The rabbit polyclonal anti-dP32 antibody and the monoclonal anti-acetylated α-tubulin antibody (Sigma) were used at a dilution of 1:1,000 and 1:200,000, respectively. For quantitation of Western blots, ECL signal intensities were measured using a Storm 860 Imaging System (GE Healthcare, Piscataway, NJ).

### Coimmunoprecipitation analysis

Coimmunoprecipitation (Co-IP) analysis was carried out essentially as described previously (Zou *et al.* 2008). Fly head lysate was prepared from transgenic flies expressing dP32-EGFP or Canton-S controls. Five hundred microliters of lysate equivalent to 50 heads and 5 μg of a rabbit polyclonal anti-GFP antibody (Invitrogen, Carlsbad, CA) were incubated with 50 μl of a 50% slurry of Protein A–Sepharose beads (Amersham Biosciences). After washing, beads were pelleted, resuspended in 20 μl of SDS sample buffer, and boiled for 3 min to elute proteins. For detection of dP32-EGFP (IP) and endogenous dP32 (co-IP), 2.5 μl or 5 μl of the 20 μl IP sample was loaded per lane, respectively. After electrophoresis, gels were processed for Western blot analysis.

### Immunocytochemistry and confocal microscopy

Immunocytochemistry and confocal microscopy were performed essentially as described previously (Kawasaki *et al.* 2011; Kawasaki and Ordway 2009). These studies employed the following primary antibodies: rabbit anti-SYT Dsyt CL1 (1:5,000) [Noreen Reist (Colorado State University, Fort Collins, CO)]; mAb nc82 anti-BRP (BRUCHPILOT) (1:50) [Erich Buchner (Universitaet Wuerzburg, Germany)]; rabbit anti-GFP (1:1,000) (Invitrogen); Cy5-conjugated rabbit anti-HRP (1:200) (Jackson Immunoresearch Laboratories, West Grove, PA). Secondary antibodies included Alexa Fluor 488-conjugated anti-mouse IgG (1:200) and Alexa Fluor 568-conjugated anti-rabbit IgG (1:200) (Invitrogen). Adult dorsal longitudinal flight muscle (DLM) neuromuscular synapse preparations were imaged using an Olympus FV1000 confocal microscope (Olympus Optical, Tokyo, Japan) with a PlanApo 60× 1.4 numerical aperture oil objective (Olympus Optical) and a z-step size of 0.2 µm. Images were obtained and processed with Fluoview software (Olympus Optical). All images shown in figures are maximum projections of two consecutive optical z-sections.

### Synaptic electrophysiology

Excitatory postsynaptic currents (EPSC) were recorded at DLM neuromuscular synapses of 3- to 5-day-old adults reared at 20°. Saline solution consisted of (in mM): 128 NaCl, 2 KCl, 4.0 MgCl_2_, 1.8 CaCl_2_, 5 HEPES, and 36 sucrose. The pH was adjusted to 7.0 using NaOH. Recordings at 33° and 36° were obtained after exposure to these temperatures for 7 min. These experiments were performed as described previously (Kawasaki and Ordway 2009).

### ATP measurements

Flies were exposed to 38°, a restrictive temperature for the *dp32^EC1^* mutant, for 3 min and then frozen in liquid nitrogen. Heads were separated from bodies as described (Zou *et al.* 2008), and 10 heads were homogenized in 100 μl of 5 M Guaindine-HCl. The homogenates were then boiled for 3 min, followed by centrifugation in a microcentrifuge at 14,000 rpm for 3 min. The supernatant was used to measure the ATP concentration using a bioluminescence assay (ATP Determination Kit; Invitrogen) according to the manufacturer’s instructions. Luminescence was measured by a luminometer (Packard Instrument Co., Meriden, CT). Briefly, the supernatant was diluted 20-fold with dH_2_O, and 20 μl of diluted supernatant was dispensed into each well of a luminometer 96-well plate, followed by mixing with 180 μl of the luciferase assay reagent. The resulting luminescence was compared with standards provided by the manufacturer to determine the ATP concentration. The relative ATP level was calculated by dividing the ATP concentration by the protein concentration, which was determined by the Bradford method (Coomassie Plus Protein Assay; Thermo Scientific, Waltham, MA). For the Bradford assay, supernatants were diluted 5-fold with dH_2_O.

### Live imaging

Imaging of TMRE, YC3.6, and GCaMP3 in the DLM presynaptic terminal was performed using an Olympus FV1000 confocal microscope with a LUM Plan 60X 1.0-NA water immersion objective (Olympus Optical) as described previously (Danjo *et al.* 2011).

For TMRE imaging, the DLM preparations were incubated in saline solution containing 5 nM TMRE for 10 min prior to image acquisition. Imaging at 36° was started after 5 min at 36°. The mitochondrial TMRE fluorescence within DLM nerve terminals was identified by colocalization with the mitochondrial marker, mito-GFP, expressed presynaptically, and the GFP-fluorescence intensity was used to normalize the TMRE signal. The GFP and TMRE imaging settings (excitation, dichroic, emission) were as follows: GFP (488 nm, SDM560, BA505-525); TMRE (543 nm, Mirror, BA560-660).

For calcium imaging using YC3.6, GCaMP3, and mito-GCaMP3, neuronal-specific expression of the membrane-associated mCD8-mRFP protein provided a red fluorescent marker for nerve terminals. The YC3.6 and GCaMP3 imaging settings (excitation, dichroic, emission) were as follows: YC3.6 (CFP, 458 nm, SDM510, BA480-495; YFP, 458 nm, Mirror, BA535-565); GCaMP3 (488 nm, SDM560, BA505-525). Time-lapse imaging was performed continuously at rates of 200 msec per frame. To minimize muscle movement during stimulation, the DLM muscle fibers were depolarized by impaling them with a sharpened tungsten wire.

Images were processed using Metavue software (Molecular Devices, Sunnyvale, CA). All image analysis was done after background subtraction. The average fluorescent intensity during a 2 sec interval before stimulus onset (F) and changes in the intensity at a given time point (ΔF) provide a measure of relative calcium concentration changes (ΔF/F). For live imaging experiments, data were obtained from at least five preparations.

### Data analysis

Microsoft (Seattle, WA) Excel was utilized to analyze numerical data and generate graphs. All data values are presented as mean ± SEM. Statistical significance was determined using the two-tailed Student *t*-test, and significance was assigned to comparisons for which *P* ≤ 0.05.

## Results

### A genetic modifier screen identifies a second chromosome enhancer of *cac^TS2^*

**Figure 1:**
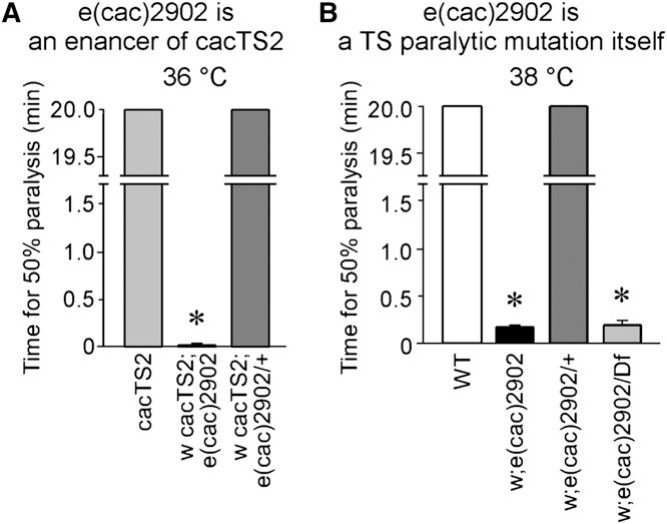
A genetic modifier screen identifies a second chromosome enhancer of *cac^TS2^*. (A) *e(cac)2902* is an enhancer of *cac^TS2^*. The double mutant, *w cac^TS2^; e(cac)2902* exhibits rapid paralysis at 36°, whereas *cac^TS2^* alone does not paralyze at this temperature. Flies heterozygous for the enhancer mutation [*w cac^TS2^; e(cac)2902/+*] exhibited a *cac^TS2^* behavioral phenotype, indicating the mutation is recessive. Behavior tests for *cac^TS2^* and *w cac^TS2^; e(cac)2902/+* were truncated after 20 min. Time for 50% paralysis in the *w cac^TS2^; e(cac)2902* double mutant was 1.3 ± 0.60 sec (n = 5). (B) *e(cac)2902* is a TS paralytic mutation itself. *e(cac)2902* in a *cac^+^* genetic background exhibits paralysis at 38°, whereas wild-type flies (WT) do not paralyze. Flies heterozygous for the *e(cac)2902* mutation exhibited a wild-type behavior, indicating the mutation is recessive. Flies carrying *e(cac)2902 in trans* to *Df(2R)14F06W-07* exhibit the *e(cac)2902* paralytic phenotype. Times for 50% paralysis in *e(cac)2902* and *e(cac)2902/Df(2R)14F06W-07* were 9.8 ± 1.77 sec (n = 5) and 11.2 ± 3.12 sec (n = 5), respectively. Behavioral tests for WT and *e(cac)2902/+* were truncated after 20 min. Asterisks mark values significantly different from control values. Here, and in subsequent figures, error bars indicate SEM, and asterisks denote statistical significance.

Our previous genetic analysis has employed phenotypic screens to identify new mutants affecting synaptic transmission. The TS presynaptic calcium channel mutant *cac^TS2^* was recovered in a screen for genetic modifiers of *comatose*, a TS synaptic transmission mutant in the N-ethylmaleimide sensitive factor 1 (dNSF1) gene (Dellinger *et al.* 2000; Kawasaki *et al.* 2000, 2002). A subsequent screen for modifiers of *cac^TS2^* identified intragenic mutations that enhance or suppress *cac^TS2^* as well extragenic enhancers in other genes (Brooks *et al.* 2003; Kawasaki *et al.* 2011). Here we extend this genetic analysis through identification of a new extragenic enhancer of *cac^TS2^*, *e(cac)2902*. On the basis that *cac^TS2^* is paralyzed rapidly at 38° but exhibits only mild motor defects at 36°, we carried out a forward genetic screen for second chromosome enhancers of the *cac^TS2^* behavioral phenotype at 36° (Figure S1). Unlike *cac^TS2^* alone, the *cac^TS2^*; *e(cac)2902* double mutant exhibits rapid paralysis at 36° (Figure 1A). This enhancer phenotype was shown to be recessive when the *e(cac)2902* mutation was made heterozygous in a *cac^TS2^* mutant background. Furthermore, after the *e(cac)2902* mutation was separated from *cac^TS2^*, it exhibited a TS paralytic phenotype by itself (Figure 1B). Thus *e(cac)2902* represents a new TS paralytic mutation identified as an enhancer of the presynaptic calcium channel mutant *cac^TS2^*.

### A synaptic phenotype in the *e(cac)2092* mutant

**Figure 2:**
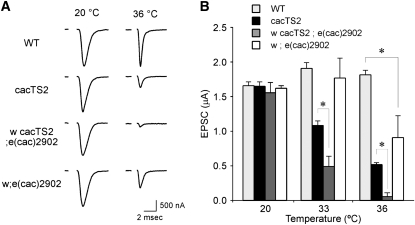
A synaptic phenotype in *e(cac)2902*. (A) Representative excitatory postsynaptic current (EPSC) recordings from dorsal longitudinal flight muscle (DLM) neuromuscular synapses of WT, *cac^TS2^*, *w cac^TS2^;e(cac)2902* double mutants, and *w;e(cac)2902* at 20° and 36°. *w cac^TS2^;e(cac)2902* exhibits a wild-type EPSC amplitude at 20°. At the restrictive temperature of 36°, the double mutant exhibited a stronger TS synaptic phenotype than *cac^TS2^* alone. *w;e(cac)2902* in a *cac^+^* genetic background produced a TS synaptic phenotype similar to that of *cac^TS2^*. Stimulation artifacts were removed for clarity. (B) Comparison of average peak EPSC amplitudes at permissive and restrictive temperatures. The respective mean EPSC amplitudes in WT, *cac^TS2^*, *w cac^TS2^;e(cac)2902*, and *w;e(cac)2902* at 20° were 1.65 ± 0.05 μA (n = 25), 1.65 ± 0.06 μA (n = 15), 1.56 ± 0.09 μA (n = 5), and 1.63 ± 0.04 μA (n = 7). The corresponding values at 33° were 2.01 ± 0.06 μA (n = 49), 1.09 ± 0.06 μA (n = 11), 0.49 ± 0.15 μA (n = 5), and 1.72 ± 0.24 μA (n = 6). The corresponding values at 36° were 1.84 ± 0.06 μA (n = 11), 0.51 ± 0.03 μA (n = 8), 0.05 ± 0.05 μA (n = 3), and 0.94 ± 0.24 μA (n = 5).

To examine whether *e(cac)2902* enhanced the *cac^TS2^* synaptic phenotype, electrophysiological recordings were performed at adult DLM neuromuscular synapses (Kawasaki and Ordway 2009). As described previously (Kawasaki *et al.* 2000) and as shown in Figure 2, synaptic transmission in *cac^TS2^* was normal at the permissive temperature of 20°. At 36°, *cac^TS2^* exhibited a marked reduction in the EPSC amplitude with respect to wild-type. In a *cac^TS2^* genetic background, the *e(cac)2902* mutation strongly enhanced the *cac^TS2^* synaptic phenotype, producing a greater EPSC amplitude reduction than in *cac^TS2^* alone (Figure 2). Furthermore, the isolated *e(cac)2902* mutation in a *cac^+^* genetic background produced a TS reduction in the EPSC amplitude similar to that observed in *cac^TS2^* (Figure 2). Both the TS synaptic and paralytic phenotypes of *e(cac)2902* recovered upon return to the permissive temperature. The preceding observations indicate that the *e(cac)2902* gene product serves an important function in synaptic transmission.

### Mapping and molecular identification of *e(cac)2092*: a new P32 mutant

**Figure 3:**
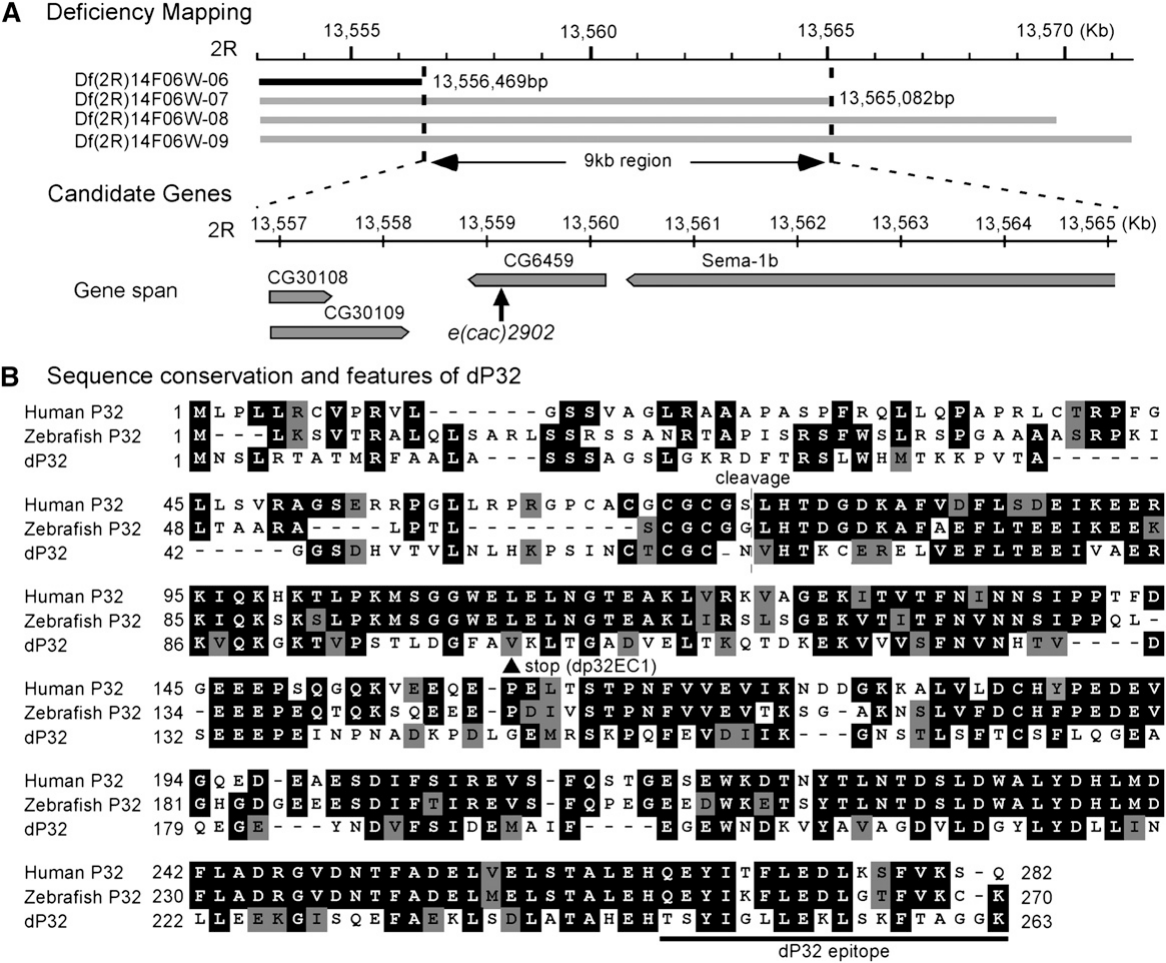
A *Drosophila* P32 mutant. (A) Meiotic and deficiency mapping placed the *e(cac)2902* mutation within a 9 kb region on the right arm of the 2^nd^ chromosome (2R). Numerical positions (in kb or bp) within the *Drosophila* genome sequence are indicated. The deleted region for each deficiency chromosome is represented by a black or gray bar. Gray indicates that the deficiency chromosome failed to complement the *e(cac)2902* behavioral phenotype when *in trans* to the *e(cac)2902* mutant chromosome. Sequencing of four candidate genes within the 9 kb region revealed a single nucleotide deletion in the CG6459 ORF resulting in a premature stop codon. C at position 303 was deleted, and thus the resulting codon changes are from [GCC GTA A] to [GCG **TAA**] (deleted nucleotide underlined; new stop codon in bold). *CG6459* is a *Drosophila* homolog of P32, and *e(cac)2902* is now termed *dp32^EC1^*. (B) Alignment of P32 homologs from human, zebrafish, and *Drosophila*. Amino acid identities and similarities are shaded in black and gray, respectively. The *dp32^EC1^* mutation introduces a premature stop codon (V102STOP; arrow head). Human P32 is synthesized as a precursor protein, of which the first 73 amino acids contain a mitochondrial targeting signal (Honoré *et al.* 1993). The mature protein is generated by site-specific cleavage after Residue 73 (broken line). The epitope for production of polyclonal antisera against dP32 is indicated (solid line). Amino acids are numbered according to their positions in the corresponding precursor proteins. Protein sequence accession numbers: human P32 (NP_001203), zebrafish P32 (XP_001331129) and dP32 (AAM11163).

To determine the location of *e(cac)2902* on the second chromosome, meiotic recombinational mapping was conducted in a *cac^TS2^* genetic background using four visible phenotypic markers, *Sternopleural* (*Sp*), *Scutoid* (*Sco*), *Lobe* (*L*), and *Pin*. Initial mapping indicated that *e(cac)2902* lies approximately 15 map units to the right of *Lobe*. Therefore, deficiency mapping of the recessive *e(cac)2092* enhancer phenotype was carried out using deficiency chromosomes covering this region (Figure 3A). Because *Df(2R)14F06W-06* complemented *e(cac)2902*, whereas *Df(2R)14F06W-07* did not, the *e(cac)2902* mutation was placed within a 9 kb region of the second chromosome containing only four candidate genes (Figure 3A).

The molecular lesion in *e(cac)2902* was identified by sequence analysis. For each of the four candidate genes, protein coding sequences were determined from the *e(cac)2902* mutant as well as the unmutagenized “parent” chromosome (Figure 3B). The only sequence change identified in the mutant was within the ORF of *CG6459*. This mutation was absent in the parent chromosome sequence and thus was likely induced by the mutagenesis. *CG6459* encodes a homolog of the evolutionarily conserved mitochondrial protein P32, which we have named dP32. Accordingly, the *e(cac)2902* mutant has been renamed *dp32^EC1^* (EC1, enhancer of *cac^TS2^* 1). The *dp32^EC1^* mutation introduces a stop codon that truncates dP32 after 101 amino acids (Figure 3) and likely represents a complete loss-of-function mutation. This observation suggests the TS paralytic phenotype of *dp32^EC1^* does not reflect a conditional disruption of dP32 activity but, rather, a role for dP32 in an inherently TS process affecting synaptic transmission.

Comparison of vertebrate and *Drosophila* P32 protein sequences revealed a high degree of conservation (Figure 3B). Mammalian P32 is targeted to mitochondria by an amino terminal targeting sequence that is cleaved to generate a mature P32 protein localized within the mitochondrial matrix. The site of P32 proteolytic cleavage appears to be conserved within dP32 (Figure 3B). Moreover, in the present study, the N-terminal domain of dP32 was shown to exhibit an equivalent mitochondrial targeting function when fused to the calcium indicator GCaMP3 (mito-GCaMP3; Figure S2).

### Western analysis and transformation rescue confirm a role for dP32 in neurotransmitter release

**Figure 4:**
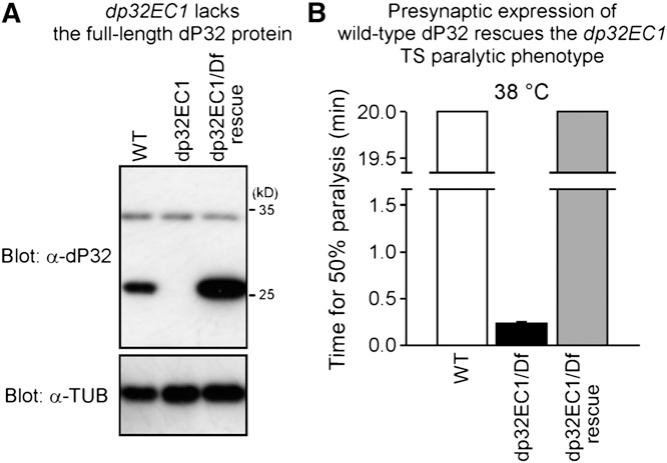
Western analysis and transformation rescue confirm that *e(cac)2902* is *dp32*. (A) Western analysis of fly head homogenates prepared from WT and *dp32^EC1^*, as well as the *dp32^EC1^* mutant expressing wild-type dP32 in the nervous system (dp32EC1/Df rescue). A prominent band at ∼26 kD is recognized by an anti-dP32 antibody. This band is absent in the mutant and elevated after neural expression of *UAS-dp32* in the *dp32^EC1^* mutant. Tubulin (TUB) was used as an internal loading control. (B) Presynaptic expression of wild-type dP32 rescues the *dp32^EC1^* paralytic phenotype. Behavioral data for WT and *dp32^EC1^/Df* are the same as those shown in Figure 1. Behavioral tests were truncated after 20 min. These results confirm that TS paralysis in *dp32^EC1^* results from disruption of dP32.

For analysis of the dP32 protein, a rabbit polyclonal antiserum was generated against a synthetic peptide corresponding to the C-terminal 18 amino acids of dP32 (Figure 3B). By Western analysis (Figure 4A), this antiserum detected dP32 in head homogenates, indicating that dP32 is expressed in the nervous system. Specificity of the antiserum was demonstrated by loss of dP32 immunoreactivity in the *dp32^EC1^* mutant (Figure 4A). These studies demonstrate that *dp32^EC1^* lacks the full-length dP32 protein as predicted from the underlying molecular lesion. Our initial characterization of dP32 expression was extended beyond the adult nervous system by conducting developmental Western analysis of whole flies, pupae, larvae, and embryos (Figure S3). dP32 expression is relatively high during embryogenesis and lower in third instar larvae. At the adult stage, more dP32 is expressed in females compared with males. This observation, along with the high levels of dP32 in early embryos, may reflect expression of dP32 in the female reproductive system. These observations are generally consistent with reported mRNA expression data (Tweedie *et al.* 2009).

To confirm that the dP32 mutation produces the observed TS paralytic and synaptic phenotypes, transformation rescue experiments were carried out using the GAL4-UAS system (Brand and Perrimon 1993). *UAS-dp32* transgenic lines were generated to express the wild-type dP32 protein, and neural expression was achieved using the *Appl-GAL4* driver line as described in our previous work [*cf*. (Kawasaki *et al.* 2011; Kawasaki and Ordway 2009)]. Transgene expression was confirmed by Western analysis (Figure 4A). Expression of wild-type dP32 in the nervous system produced clear rescue of the *dp32^EC1^* TS paralytic phenotype (Figure 4B), demonstrating an important function for dP32 in the nervous system. Furthermore, electrophysiological recordings confirmed that transgenic expression of wild-type P32 rescues the *dp32^EC1^* synaptic phenotype (Figure S4).

### Localization of dP32 in mitochondria at DLM neuromuscular synapses

**Figure 5:**
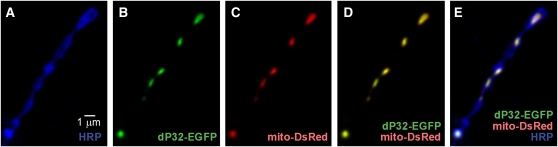
Localization of dP32 in mitochondria at DLM neuromuscular synapses. (A–E) Confocal immunofluorescence and native EGFP and DsRed fluorescence images of adult DLM neuromuscular synapses exhibiting neuronal (presynaptic) expression of dP32-EGFP and mito-DsRed. Mito-DsRed serves as a marker for mitochondria. Anti-HRP labels the neuronal plasma membrane. Colocalization of dP32-EGFP and mito-DsRed demonstrates a mitochondrial distribution for dP32.

Previous studies indicate that dP32 proteins are localized to mitochondria (Dedio *et al.* 1998; Muta *et al.* 1997; Seytter *et al.* 1998; but see also Fogal *et al.* 2008; Ghebrehiwet *et al.* 2001; Majumdar *et al.* 2002; Matthews and Russell 1998), and the mitochondrial targeting sequence of human P32 has been characterized (Honoré *et al.* 1993). Although the anti-dP32 antibody is suitable for Western analysis and demonstrated clear expression of dP32 in the nervous system, the same antibody produced no signal above background in immunocytochemical studies of dP32. Thus, UAS transgenic lines were generated to express a form of dP32 tagged with EGFP at the C-terminus, dP32-EGFP. Expression of dP32-EGFP in the nervous system of *dp32^EC1^* mutants produced rescue of TS paralysis (Figure S5), indicating that dP32-EGFP retains its function. These developments permitted analysis of dP32 distribution in neurons. Following neural expression of dP32-EGFP, imaging native dP32-EGFP fluorescence at DLM neuromuscular synapses demonstrated clear mitochondrial localization (Figure 5).

### Biochemical analysis of dP32

**Figure 6:**
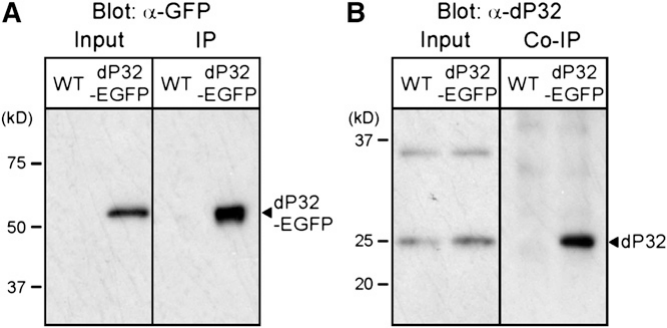
Homomultimeric interactions of dP32: dP32-EGFP and endogenous dP32 coimmunoprecipitate from fly head lysate. An anti-GFP antibody was used to precipitate dP32-EGFP from head lysate of flies expressing dP32-EGFP in the nervous system or control flies (WT) lacking the transgene. Immunoprecipitation (IP) of dP32-EGFP (A) and co-IP of endogenous dP32 (B) was demonstrated by Western blotting using anti-GFP and anti-dP32 antibodies, respectively. Specific co-IP of dP32 was observed only in samples expressing dP32-EGFP, indicating homomultimeric association of dP32-EGFP and endogenous dP32 subunits.

Understanding the mechanisms of dP32 function in mitochondria and synaptic transmission will require defining its molecular interactions. Previous biochemical and structural studies have established that P32 exhibits a homotrimeric quaternary structure (Ghebrehiwet *et al.* 1994; Jiang *et al.* 1999). As a starting point for biochemical analysis of dP32 in *Drosophila*, we examined whether analogous homomeric interactions occur in neural tissue. These studies achieved specific immunoprecipitation of the dP32-EGFP fusion protein using an anti-GFP antibody. Tissue samples from wild-type flies lacking expression of the dP32-EGFP transgene served as controls. In fly head homogenates, specific immunoprecipitation of dP32-EGFP was observed (Figure 6A). These immunoprecipitates were examined for co-IP of endogenous dP32 as an indication of homomeric interactions of dP32. Specific co-IP of endogenous dP32 was observed in samples from flies expressing dP32-EGFP but not in wild-type control samples (Figure 6B). These results confirm homomeric interactions of dP32.

### Preservation of presynaptic organization in the *dp32^EC1^* mutant

As a first step in examining the possible functions of dP32 in synaptic transmission, basic synaptic morphology was assessed in the *dp32^EC1^* mutant. Although it was considered unlikely that enhancement of the *cac^TS2^* synaptic phenotype by *dp32^EC1^* was associated with changes in the morphology of presynaptic terminals, immunocytochemical analysis of DLM neuromuscular synapses from wild-type or the *cac^TS2^;dp32^EC1^* double mutant was performed using markers for the neuronal plasma membrane, the active zone, and synaptic vesicles (Figure S6). These studies indicated no obvious changes in basic synapse morphology, including the distributions of active zones and synaptic vesicle clusters. These observations are consistent with the lack of a synaptic phenotype at a permissive temperature and suggest that loss of dP32 protein produces a TS functional defect at morphologically normal synapses.

**Figure 7:**
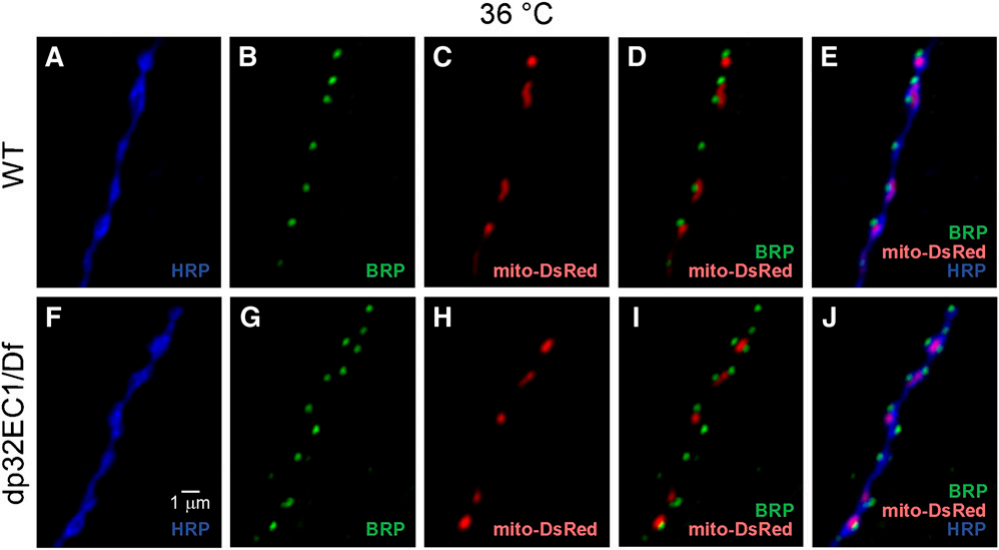
Wild-type presynaptic distribution of mitochondria in the *dp32^EC1^* mutant. Confocal images of adult DLM neuromuscular synapses expressing mito-DsRed in WT (A–E) and the *w;dp32^EC1^/ Df(2R)14F06W-07* mutant (dp32EC1) **(**F–J**)**. Dissected preparations were exposed to a restrictive temperature of 36° for 7 min and then fixed and processed for immunocytochemistry. Mitochondria were visualized by native mito-DsRed fluorescence. Anti-HRP and anti-BRP label the neuronal plasma membrane and presynaptic active zones, respectively. The presence of presynaptic mitochondria adjacent to active zones was observed in both wild-type and mutant nerve terminals.

Finally, synaptic organization was examined with respect to the distribution of presynaptic mitochondria, which is altered in several *Drosophila* mutants (Guo *et al.* 2005; Stowers *et al.* 2002; Verstreken *et al.* 2005). To investigate whether dP32 plays a role in mitochondrial transport or localization, immunocytochemical analysis of mito-DsRed localization with respect to an active zone marker was examined at DLM neuromuscular synapses of wild-type and *dp32^EC1^* after exposure to a restrictive temperature of 36°. At both wild-type and mutant synapses, mitochondria were associated with most active zones (Figure 7), indicating proper transport and localization of presynaptic mitochondria in the *dp32^EC1^* mutant.

### Possible functional roles of dP32 in synaptic transmission

The preceding findings indicate that basic synapse morphology and localization of presynaptic mitochondria is normal in the *dp32^EC1^* mutant. These findings, together with the observed mitochondrial localization of dP32, suggest that dP32 plays an important physiological role in mitochondria to support the function of established synapses. Three key aspects of mitochondrial function were considered: the mitochondrial membrane potential, ATP production, and mitochondrial calcium signaling.

### Mitochondrial membrane potential and ATP synthesis

The membrane potential maintained across the inner mitochondrial membrane participates in several aspects of mitochondrial function and is essential for ATP production. As described in the following text, these critical aspects of mitochondrial function might be altered in the dP32 mutant and lead to disruption of synaptic transmission.

#### The *dP32^EC1^* mutant exhibits a wild-type mitochondrial membrane potential (ΔΨm):

**Figure 8:**
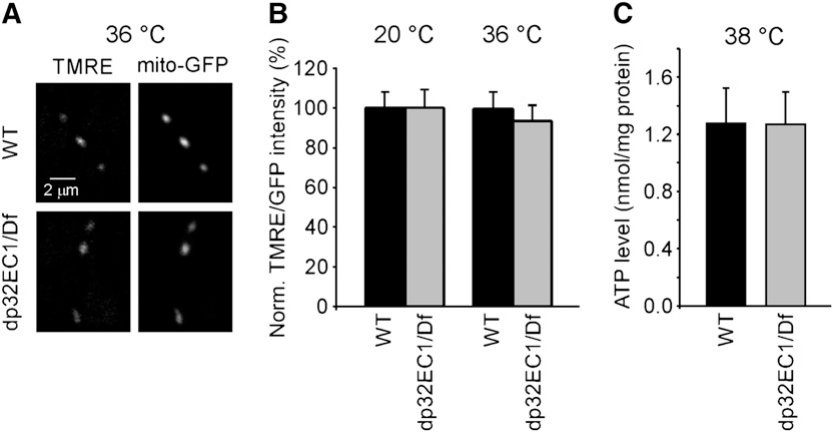
*dp32^EC1^* mutant retains wild-type mitochondrial membrane potential (ΔΨm) and ATP production. (A, B) ΔΨm measurements using tetramethylrhodamine ethyl ester perchlorate (TMRE) and GFP targeted to the mitochondrial matrix (mito-GFP). (A) Examples of live confocal images of TMRE at DLM neuromuscular synapses exhibiting neuronal expression of *UAS-mito-GFP* in a WT or *dp32^EC1^* genetic background. Mito-GFP serves as a mitochondrial marker within presynaptic terminals. (B) *dp32^EC1^* retains wild-type ΔΨm. The ratio of TMRE to GFP (TMRE/GFP) from the same region of interest was used to represent the relative ΔΨm, which should be independent of mitochondrial size, shape, and orientation. The relative ΔΨm from *dp32^EC1^* is shown as a mean percentage of that from WT. The ΔΨm in *dp32^EC1^* is not significantly different from WT at either temperature. The respective ratios of TMRE to GFP at WT and *dp32^EC1^* synapses were 1.32 ± 0.11 (n = 26) and 1.30 ± 0.11 (n = 23) at 20°. The corresponding values at 36° were 0.60 ± 0.06 (n = 22) and 0.56 ± 0.05 (n = 24). (C) Head homogenates from *dp32^EC1^* contain wild-type levels of ATP. Head homogenates prepared from WT and *dp32^EC1^* flies were exposed to 38° for 10 min and subjected to a luciferin-luciferase ATP detection assay. The relative ATP levels were calculated by dividing the luminescence by the total protein concentration. The relative ATP levels in WT and *dp32^EC1^* were 1.28 ± 0.24 nmol/mg protein (n = 5) and 1.27 ± 0.23 nmol/mg protein (n = 5), respectively, and not significantly different.

Previous studies have disrupted the membrane potential of presynaptic mitochondria and examined its contribution to synaptic transmission (Billups and Forsythe 2002). This work revealed that loss of ΔΨm produces a stimulation-dependent reduction in neurotransmitter release. A possible role for dP32 in maintaining ΔΨm was examined using the fluorescent indicator tetramethylrhodamine ethyl ester (TMRE). Accumulation of TMRE in mitochondria is related to ΔΨm and may be used to determine changes in ΔΨm as described previously (Twig *et al.* 2006). The relative mitochondrial ΔΨm was examined by comparing fluorescence intensities at wild-type and *dP32^EC1^* DLM neuromuscular synapses after exposure to TMRE for 10 min. As in previous work (Twig *et al.* 2006), the mitochondrial TMRE fluorescence intensity was normalized to a GFP-based mitochondrial marker, mito-GFP, to control for differences in the size and shape of the mitochondria in each optical section. Studies carried out at both 20° and the restrictive temperature of 36° indicated that *dP32^EC1^* exhibits a wild-type ΔΨm (Figure 8, A and B).

#### Head homogenates from *dP32^EC1^* contain wild-type levels of ATP:

Mitochondrial ATP production plays a key role in providing energy for synaptic function. Previous work in *Drosophila* indicates that stress sensitive B, a mitochondrial protein, contributes to synaptic transmission through the generation of ATP (Rikhy *et al.* 2003; Trotta *et al.* 2004). To examine whether dP32 functions in this process, ATP levels were measured in homogenized head tissue from wild-type and *dP32^EC1^* mutant flies. These studies employed a luciferin-luciferase ATP assay as described previously (Al-Anzi *et al.* 2009; Liu *et al.* 2007; Park *et al.* 2006). ATP measurements carried out on homogenates from flies exposed to the restrictive temperature of 38° indicated no significant difference between wild-type controls and the *dP32^EC1^* mutant (Figure 8C). These observations represent a global analysis of brain tissue, including central synapses. Although they provide strong evidence against a role for dP32 in ATP production, they cannot exclude the possibility of local changes in ATP.

The preceding findings indicate that both mitochondrial ΔΨm and ATP production persist after loss of dP32 function, and thus, that the role of dP32 in synaptic transmission does not involve these critical aspects of mitochondrial function.

### Possible role for dP32 in mitochondrial calcium signaling

Because mitochondria sequester and release calcium within the presynaptic terminal, they can play important roles in regulation of cytosolic calcium, calcium-dependent neurotransmitter release, and synaptic plasticity (Billups and Forsythe 2002; David and Barrett 2003; Tang and Zucker 1997; Yang *et al.* 2003). In light of the observed role for dP32 in neurotransmitter release, a function for this protein in presynaptic calcium signaling was examined.

#### *dP32^EC1^* exhibits a conditional increase in presynaptic calcium with respect to wild-type:

**Figure 9:**
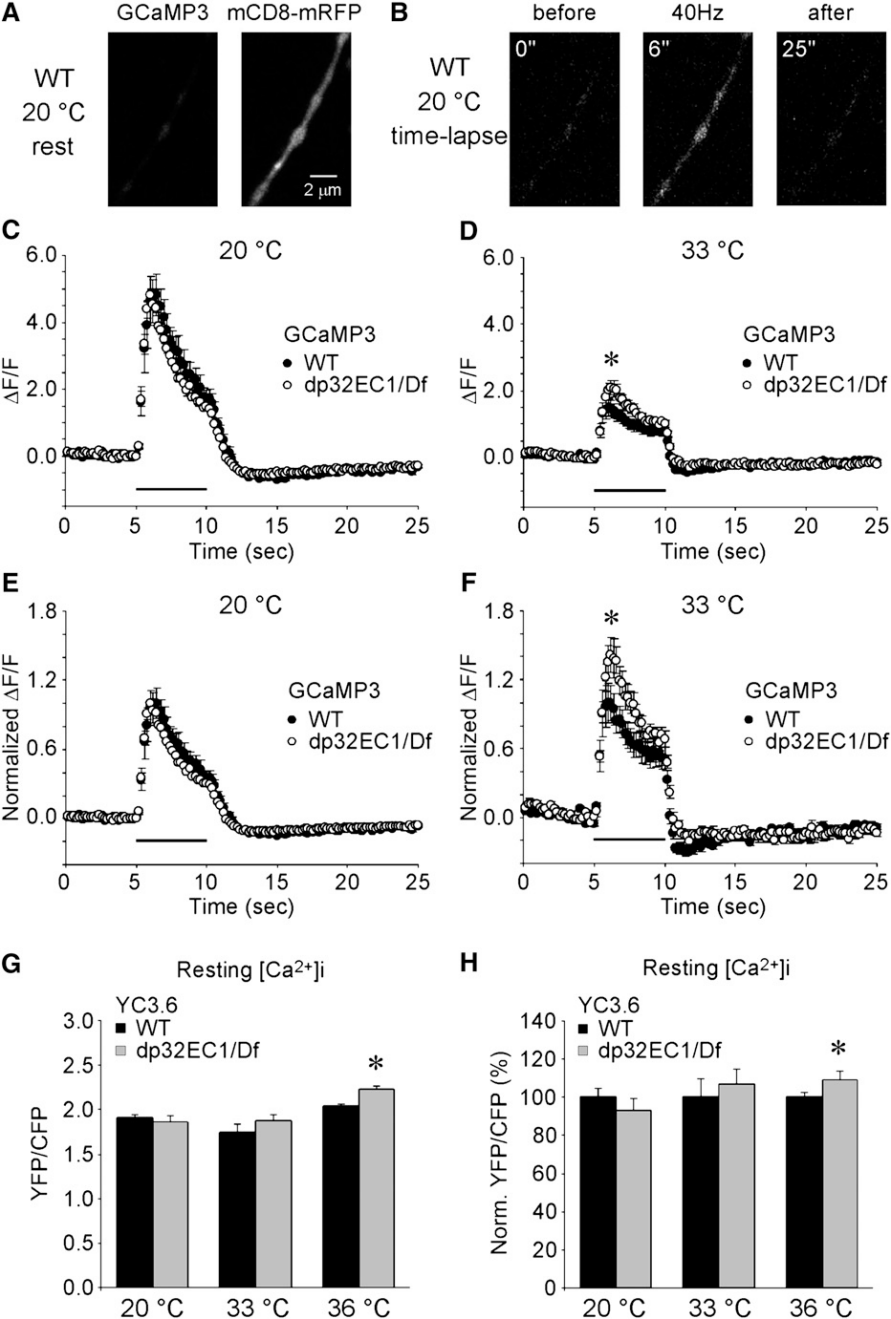
*dp32^EC1^* exhibits conditional defects in presynaptic calcium signaling. (A–F) Imaging of cytosolic calcium transients within DLM neuromuscular presynaptic terminals expressing the calcium indicator GCaMP3. (A) A representative image of GCaMP3 at a resting WT synapse at 20°. Neuronal expression of the membrane-associated mCD8-mRFP protein provided a red fluorescent marker for nerve terminals. (B) Example images of GCaMP3 at WT synapses before, during, and after DLM motor axon stimulation (40 Hz for 5 sec) at 20°. Times indicated are relative to the start of the time-lapse imaging, and axon stimulation was initiated at 5 sec. (C–F) Fluorescence changes (ΔF/F) were examined at WT and *dp32^EC1^* (dp32EC1/Df) synapses. (C) At 20°, DLM motor axon stimulation at 40 Hz for 5 s (bar above X axis) produced a similar increase in cytosolic calcium at WT (n = 11) and *dp32^EC1^* (n = 17) synapses. In contrast, the same stimulation protocol at 33° (D) elicited a larger calcium transient in *dp32^EC1^* (n = 13) than in WT (n = 14). The peak ΔF/F in *dp32^EC1^* (3.09 ± 0.22) was significantly increased with respect to WT (2.48 ± 0.22). The data shown in C and D were normalized to the maximum ΔF/F in WT at each temperature and replotted in E and F, respectively. Asterisks in D and F indicate that the maximum ΔF/F in *dp32^EC1^* is significantly greater than that in WT. (G, H) Imaging of relative resting presynaptic calcium concentrations within DLM neuromuscular presynaptic terminals expressing the ratiometric calcium indicator Cameleon YC3.6. The EYFP/ECFP emission ratio (YFP/CFP) was used to assess the relative resting presynaptic calcium concentrations at WT and *dp32^EC1^* (dp32EC1/Df) synapses at 20°, 33°, and 36° (G). (H) The data shown in G were normalized to the mean YFP/CFP ratio in WT at each temperature. At restrictive temperatures, resting calcium was increased in *dp32^EC1^* with respect to WT. As a percentage of WT, the resting calcium concentration at *dp32^EC1^* synapses at 20° was 93 ± 4% (WT, n = 17; *dp32^EC1^*, n = 11). The corresponding values at 33° and 36° were 107 ± 4% (WT, n = 12; *dp32^EC1^*, n = 12) and 109 ± 2% (WT, n = 13; *dp32^EC1^*, n = 9), respectively.

Measurement of cytosolic calcium transients elicited by synaptic stimulation was carried out through transgenic expression of the GFP-based calcium indicator GCaMP3 in the nervous system (Tian *et al.* 2009) (Figure 9, A–F). This approach was used to compare presynaptic calcium transients at wild-type or *dP32^EC1^* mutant DLM neuromuscular synapses. At 20°, 40 Hz stimulation (200 pulses) of wild-type and mutant synapses produced similar calcium transients (Figure 9, C and E). As described previously for GCaMP1.3 and GCaMP1.6 measurements of presynaptic calcium transients (Reiff *et al.* 2005), synaptic stimulation elicits an initial peak in the fluorescence intensity, followed by a gradual decline during the stimulation train, and an undershoot phase after stimulation. To examine a possible conditional phenotype in *dP32^EC1^*, analogous calcium imaging experiments were carried out at 33°. At this temperature, *dP32^EC1^* enhances the *cac^TS2^* synaptic phenotype (Figure 2) but does not have a strong synaptic phenotype in a *cac^+^* background. Attempts to image calcium transients at 36°, at which *dP32^EC1^* has a clear synaptic phenotype in the absence of *cac^TS2^*, were unsuccessful due to motion artifacts in the DLM neuromuscular synapse preparation at this temperature. Nonetheless, a conditional *dP32^EC1^* phenotype was observed at 33°. At this temperature, the calcium transient elicited by 40 Hz stimulation was increased in *dP32^EC1^* with respect to wild-type (Figure 9, D and F). This observation was surprising, given that *dP32^EC1^* produces a conditional decrease in calcium-triggered neurotransmitter release. These findings indicate a role for dP32 in regulating presynaptic calcium transients during synaptic activity and suggest that dP32 functions in mitochondrial calcium signaling (see *Discussion*).

The preceding results raised the possibility that elevated resting presynaptic calcium in *dP32^EC1^* might impair neurotransmitter release. Thus, additional experiments were carried out to assess resting presynaptic calcium concentrations. These studies employed the ratiometric calcium indicator yellow cameleon 3.6 (YC3.6) (Nagai *et al.* 2004) to determine relative resting presynaptic calcium concentrations at wild-type and *dP32^EC1^* synapses. These experiments were carried out at 20°, 33°, and 36°, the latter feasible because of the short time-lapse imaging periods (5 sec) required for imaging resting calcium. At 20°, no significant difference was observed in the resting presynaptic calcium concentration at wild-type and *dP32^EC1^* synapses (Figure 9, G and H). In contrast, resting calcium was increased in *dP32^EC1^* relative to wild-type at elevated temperatures (Figure 9, G and H). This relative increase in resting calcium in *dP32^EC1^* was significant at 36°. It is interesting to consider whether this conditional increase in cytosolic calcium in *dP32^EC1^* might impair evoked neurotransmitter release (see *Discussion*).

## Discussion

We conducted a genetic screen for synaptic transmission mutants in *Drosophila* and isolated a new mutation in a *Drosophila* homolog of the mitochondrial protein P32, which represents the first P32 mutation in a multicellular organism. Although P32 is highly conserved and has been studied extensively, its physiological function in mitochondria remains unknown. This new mutant, referred to as *dP32^EC1^*, exhibited a temperature-sensitive (TS) paralytic behavioral phenotype. Moreover, electrophysiological analysis at adult neuromuscular synapses revealed a TS reduction in neurotransmitter release, indicating that dP32 serves an important function in synaptic transmission. Our immunocytochemical analysis has shown that dP32 is located within presynaptic mitochondria, which are known to be important in ATP production and calcium signaling at synapses. Furthermore, the basic molecular and structural organization of synapses appears to be normal in the dP32 mutant, suggesting a direct role for this protein in synaptic function. At the molecular level, our biochemical studies indicated conserved homomultimeric interactions of dP32 subunits. Finally, assessment of presynaptic mitochondrial function was examined in the dP32 mutant through measurement of ATP levels and imaging studies of mitochondrial membrane potential and presynaptic calcium. This work indicated that mitochondrial ATP production and membrane potential in the dP32 mutant resembled wild-type, whereas the mutant exhibited a TS increase in both resting and evoked presynaptic calcium concentration. Taken together, the preceding findings reveal a role for dP32 in synaptic transmission and mitochondrial regulation of presynaptic calcium.

### Molecular properties of dP32

Mitochondrial localization of P32 proteins involves an N-terminal targeting domain that is cleaved from the mature targeted protein (Honoré *et al.* 1993). Comparison of *Drosophila* and vertebrate P32 sequences indicates conservation of the proteolytic cleavage site in dP32 (Figure 3B). In the present study, an equivalent targeting function for the N-terminal domain of dP32 was demonstrated through its ability to mediate mitochondrial targeting. When the first 71 amino acids of dP32, including the proteolytic cleavage site, was fused to GCaMP3, this fusion protein (mito-GCaMP3) was efficiently targeted to mitochondria (see *Materials and Methods* and Figure S2). Although only modest sequence conservation was observed between the N-terminal domains of dP32 and vertebrate P32 proteins (Figure 3B), previous studies suggest that mitochondrial targeting domains vary in amino acid sequence but often share an amphipathic helical structure [reviewed in Chacinska *et al.* (2009)].

Structural studies have established that P32 is a homotrimer in which monomers are arranged around a central pore in a donut-like structure. In the present study, homomultimerization of dP32 subunits was demonstrated in co-immunoprecipitation experiments (Figure 6). The trimeric structure of P32 exhibits a highly asymmetric charge distribution that creates a concentration of negatively charged residues along one side of the donut, raising the possibility that P32 may participate in calcium binding within the mitochondrial matrix (Jiang *et al.* 1999). Notably, five residues that are spatially clustered to form a pocket on the negatively charged side of human P32, Glu-89, Leu-231, Asp-232, Glu-264, and Tyr-268, are identical in the *Drosophila* protein. Further genetic analysis may address the importance of these clustered residues in dP32 function at synapses.

### Possible mechanism of dP32 function in neurotransmitter release

In the present study, several possible mechanisms of dP32 function in mitochondria and synaptic transmission were considered and investigated, most notably possible roles in supporting mitochondrial membrane potential, ATP production, and presynaptic calcium signaling. Among these, our observations favor a function for dP32 in mitochondrial mechanisms regulating presynaptic calcium. Although neurotransmitter release was reduced at restrictive temperatures in *dP32^EC1^*, the presynaptic calcium concentration was increased both at rest and in response to synaptic stimulation. It is of interest to consider why the increase in presynaptic calcium in *dP32^EC1^* is TS in what appears to be a complete loss-of-function mutant. Previous studies at *Drosophila* larval neuromuscular synapses at elevated temperatures have observed a TS increase in resting cytosolic calcium and associated inhibition of neurotransmitter release (Klose *et al.* 2009). This calcium increase was enhanced by pharmacological inhibition of presynaptic calcium clearance mechanisms or genetic removal of presynaptic mitochondria, but it remained dependent on temperature. The present findings may reflect a similar TS process involving calcium-dependent inhibition of neurotransmitter release and dP32-dependent mitochondrial mechanisms. Efforts to further address these mechanisms were pursued by employing a calcium indicator targeted to the mitochondrial matrix, mito-GCaMP3 (see *Materials and Methods* and Figure S2). Although this approach was successful for imaging mitochondrial calcium transients elicited by motor axon stimulation in both WT and *dP32^EC1^* at 20° (Figure S7), robust calcium transients could not be observed in either genotype when the temperature was increased to the restrictive temperatures of 33° or 36°.

The preceding observations suggest that sustained elevation of presynaptic calcium in the dP32 mutant may lead to reduced neurotransmitter release. Such a calcium-dependent mechanism has been reported previously in the squid giant synapse and attributed to calcium-dependent adaptation of the neurotransmitter release apparatus (Hsu *et al.* 1996). Understanding the precise mechanism by which loss of dP32 impairs neurotransmitter release will require further investigation. One interesting question is how the absence of dP32 in the mitochondrial matrix leads to increased presynaptic calcium and whether this reflects the putative calcium binding capacity of this protein. Finally, while the present study is focused on the newly discovered role for P32 in neurotransmitter release, the resulting research materials are expected to facilitate *in vivo* analysis of P32 function in a broad range of biological processes.

## Supplementary Material

Supporting Information
