# Supplementary material for: Genetic Analysis in Drosophila Reveals a Role for the Mitochondrial Protein P32 in Synaptic Transmission
Source: G3 (Bethesda). 2012 Jan 1;2(1):59–69. doi: 10.1534/g3.111.001586 (PMC3276185; doi:10.1534/g3.111.001586)
Supplement: Supporting Information [file supp_2_1_59__index.html]

Supporting Information 

# Genetic Analysis in *Drosophila* Reveals a Role for the Mitochondrial Protein P32 in Synaptic Transmission

## Supporting Information for Lutas *et al.*, 2012

**Files in this Data Supplement:**

- Supporting Information - Figures S1-S7 (PDF, 1.5 MB)
- Figure S1 - A Genetic Screen for Second Chromosome Modifiers of *cacTS2* (PDF, 136 KB)
- Figure S2 - Localization of dP32 in Mitochondria at DLM Neuromuscular Synapses (PDF, 384 KB)
- Figure S3 - Developmental expression of dP32 (PDF, 164 KB)
- Figure S4 - Presynaptic Expression of Wild-Type dP32 Rescues the *dp32EC1* Synaptic Phenotype (PDF, 88 KB)
- Figure S5 - Presynaptic Expression of dP32-EGFP Rescues the *dp32EC1* Paralytic Phenotype (PDF, 88 KB)
- Figure S6 - Preservation of Presynaptic Composition in the *dp32EC1* Mutant (PDF, 456 KB)
- Figure S7 - Mitochondrial Calcium Transients within DLM Neuromuscular Presynaptic Terminals (PDF, 296 KB)
