# Supplementary material for: Genetic Analysis in Drosophila Reveals a Role for the Mitochondrial Protein P32 in Synaptic Transmission
Source: G3 (Bethesda). 2012 Jan 1;2(1):59–69. doi: 10.1534/g3.111.001586 (PMC3276185; doi:10.1534/g3.111.001586)
Supplement: Supporting Information [file supp_2.1.59_001586SI.pdf]

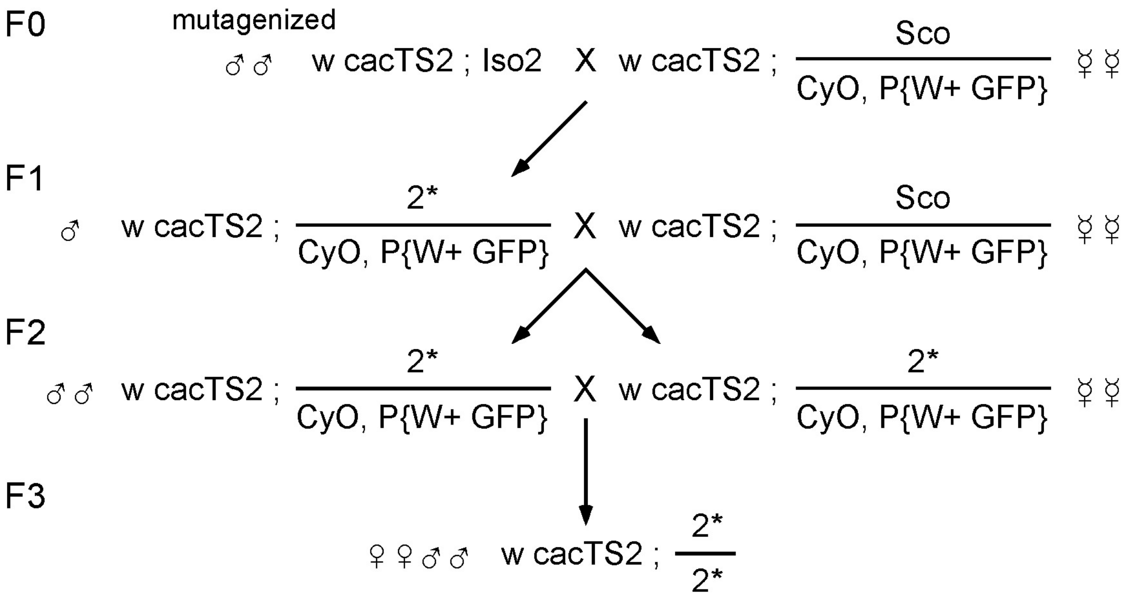

Figure S1 A Genetic Screen for Second Chromosome Modifiers of *cac*^{TS2}. *cac*^{TS2} males with an isogenized second chromosome were exposed to the mutagen, ethylmethane sulphonate (EMS). Mutagenized males were mated with *cac*^{TS2} females carrying the visible second chromosome marker, *Scutoid* (*Sco*), *in trans* to a *Curly of Oster* (*CyO*) balancer chromosome carrying a GFP transgene marked with *w*$^{+}$. The F1 male progeny were backcrossed to F0 females. After mating F2 heterozygous siblings, F3 flies homozygous for a mutagenized second chromosome in a *cac*^{TS2} genetic background were screened for altered *cac*^{TS2} behavior at 36°C. * ; mutagenized chromosome

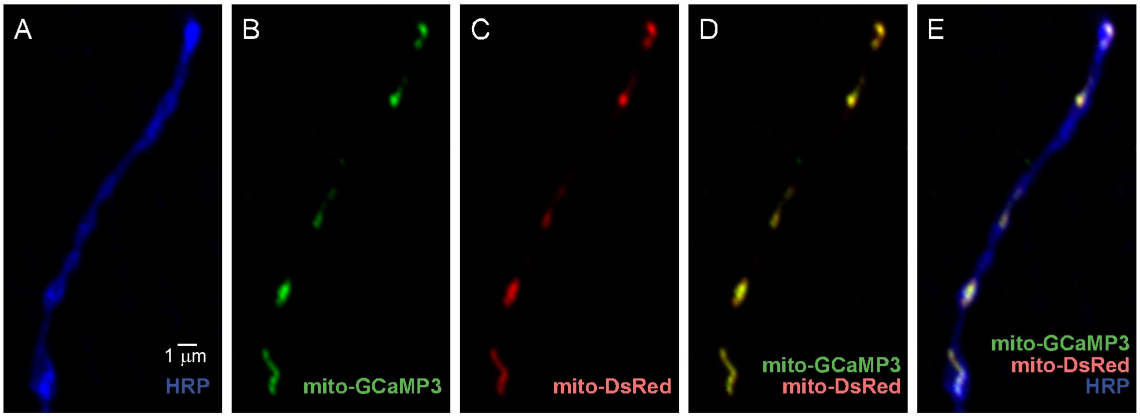

Figure S2 Localization of dP32 in Mitochondria at DLM Neuromuscular Synapses. Confocal immunofluorescence and native DsRed fluorescence images of adult DLM neuromuscular synapses exhibiting neuronal expression of mito-GCaMP3 and mito-DsRed. Mito-GCaMP3 consists of the first 71 amino acids of dP32 fused to the N-terminus of the calcium indicator, GCaMP3. Mito-GCaMP3 was recognized by an anti-GFP antibody. Mito-DsRed, which includes the previously characterized mitochondrial targeting domain of human COX8 (see Methods), serves as a mitochondrial marker. Anti-HRP labels the neuronal plasma membrane. Colocalization of mito-GCaMP3 and mito-DsRed demonstrates that mito-GCaMP3 was efficiently targeted to mitochondria.

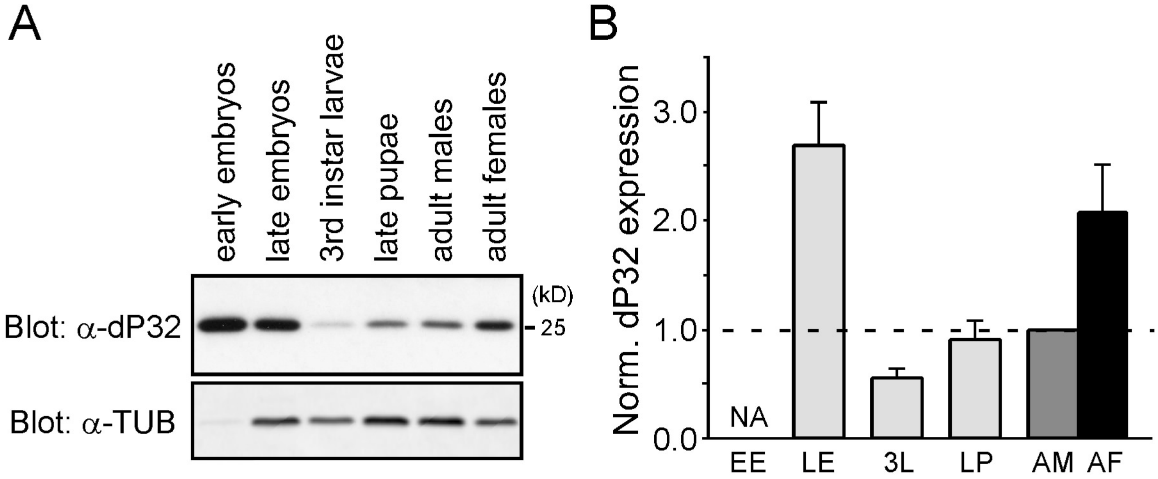

Figure S3 Developmental expression of dP32. Western analysis of whole animal homogenates prepared from WT at different stages of development: early embryos (EE), 0-4 hr after egg laying; late embryos (LE), 20-24 hr; third instar larvae (3L); late pupae (LP); adult males (AM) and females (AF). (A) Endogenous dP32 was recognized by an anti-dP32 antibody. Tubulin (TUB) was used as an internal loading control. (B) Comparison of average expression of dP32 at different stages of development obtained from three independent experiments. In each experiment, the dP32 signals at different stages were first divided by the corresponding Tubulin signal and the resulting values were normalized to those from adult male samples. The respective mean expression levels in LE, 3L, LP and AF were 2.68 ± 0.39, 0.56 ± 0.07, 0.91 ± 0.17 and 2.08 ± 0.44. NA (not applicable) indicates that quantitative analysis of the EE samples was not performed due to low expression of Tubulin at this stage.

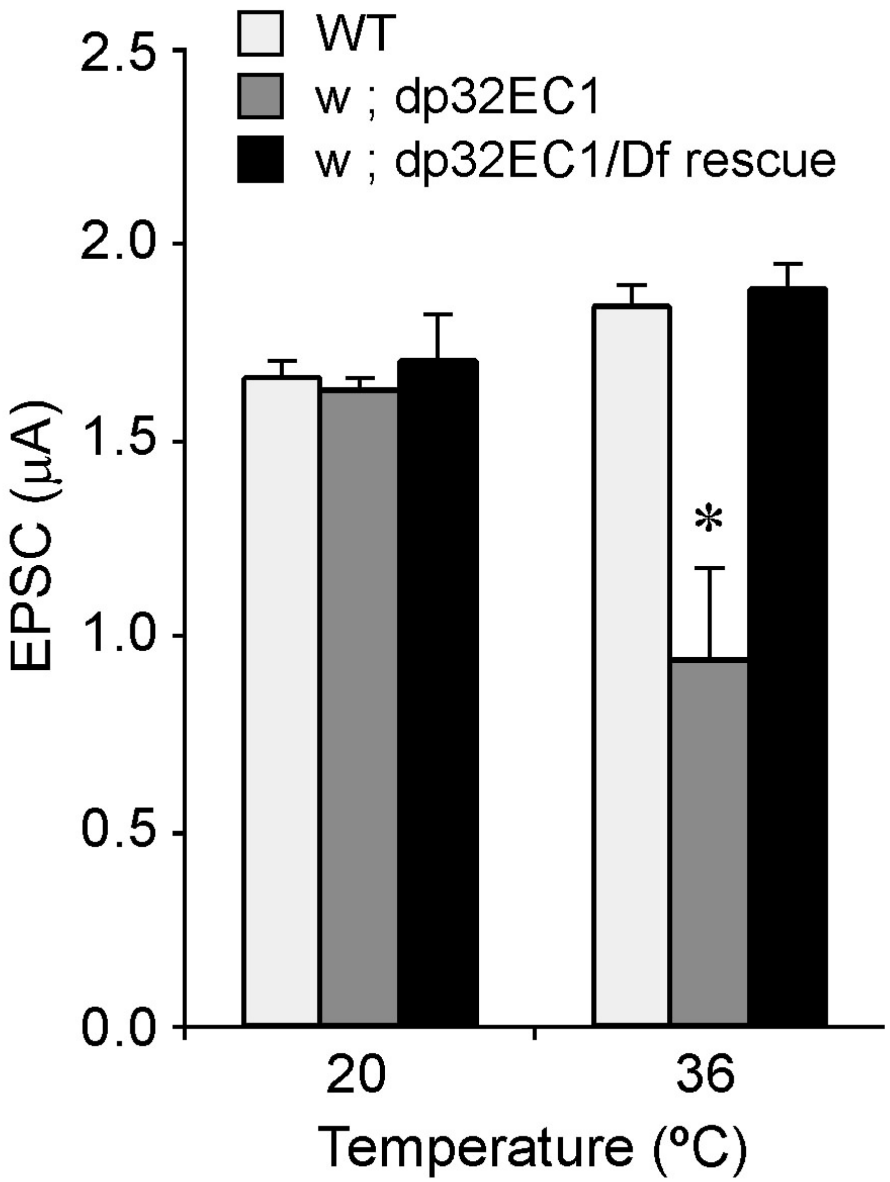

Figure S4 Presynaptic Expression of Wild-Type dP32 Rescues the *dp32*^{EC1} Synaptic Phenotype. Mean EPSC amplitudes in WT and the *dp32*^{EC1} mutant, as well as the *dp32*^{EC1} mutant expressing wild-type dP32 in the nervous system (dp32EC1/Df rescue). At 36°C, the EPSC amplitude in *dp32*^{EC1}*/Df* rescue [1.89 ± 0.07 µA (n = 5)] was not significantly different from that in WT (p = 0.65). The data for WT and *dp32*^{EC1}are the same as in Figure 2.

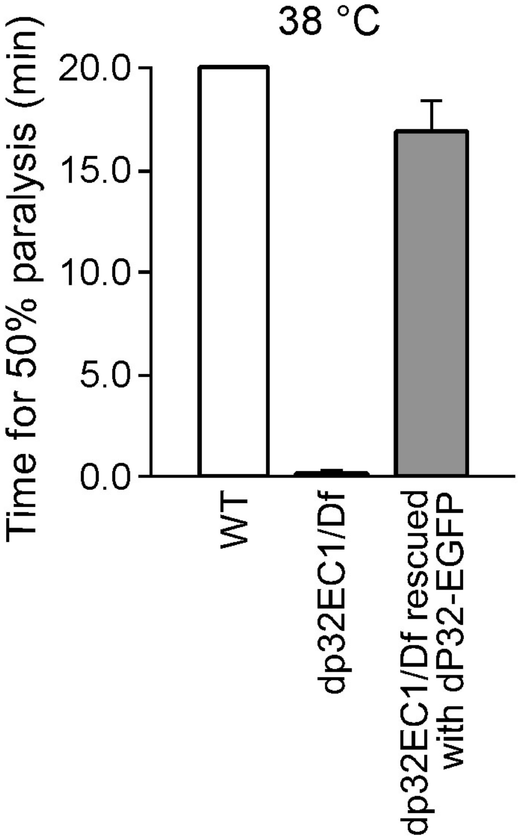

Figure S5 Presynaptic Expression of dP32-EGFP Rescues the *dp32*^{EC1} Paralytic Phenotype. Rescue of TS paralysis in the *dp32*^{EC1} mutant was examined at 38°C. The time for 50% paralysis was increased from 11.2 ± 3.12 sec (n = 5) in *dp32*^{EC1} (dp32EC1/Df) to 16.9 ± 1.48 min (n = 5) in *dp32*^{EC1} expressing dP32-EGFP in the nervous system (dp32EC1/Df rescued with dP32-EGFP). The WT behavioral test was truncated after 20 minutes. These results demonstrate that the dP32-EGFP fusion protein retains its function. The data for WT and *dp32*^{EC1}are the same as in Figure 1B.

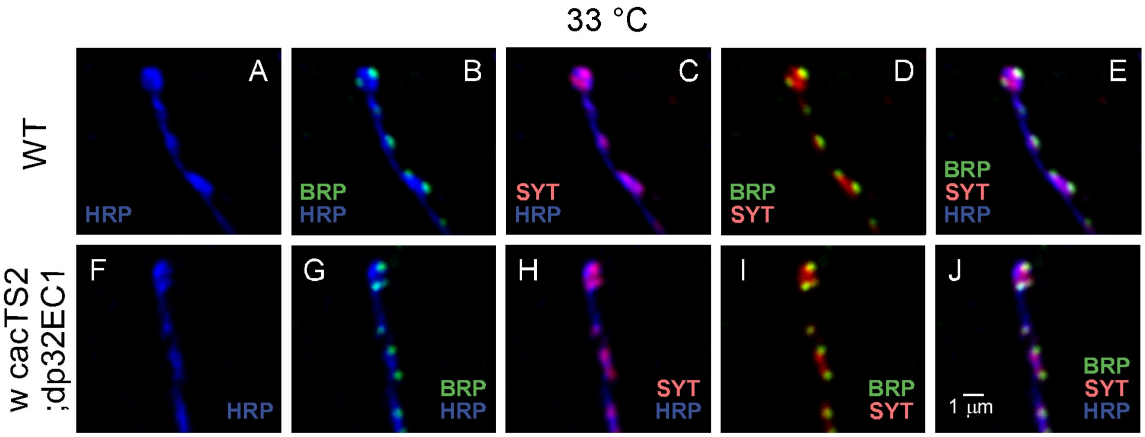

Figure S6 Preservation of Presynaptic Composition in the *dp32*^{EC1} Mutant. Confocal immunofluorescence images of adult DLM neuromuscular synapses in WT and *w cac*^{TS2}*; dp32*^{EC1} double mutants. Dissected preparations were exposed to a restrictive temperature of 33°C for 10 minutes and then fixed for processing. At this temperature, double mutant synapses exhibit a severe reduction in EPSC amplitude (Figure 2)]. Anti-HRP labels the neuronal plasma membrane such that the motor axon and its presynaptic boutons (swellings) are visualized. Anti-BRP labels presynaptic active zones. Anti-SYNAPTOTAGMIN (SYT) labels synaptic vesicles.

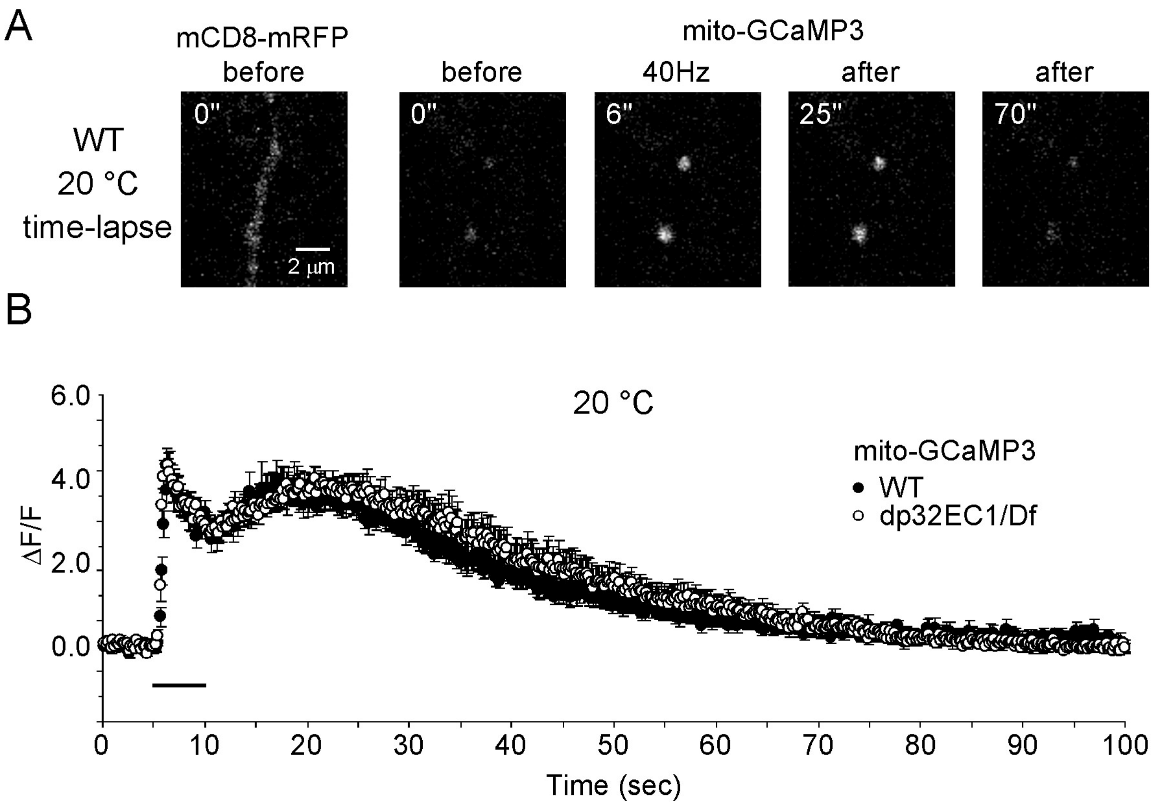

Figure S7 Mitochondrial Calcium Transients within DLM Neuromuscular Presynaptic Terminals. Imaging of mitochondrial calcium transients was performed at WT and *dp32*^{EC1} (dp32EC1/Df) synapses expressing the GCaMP3 targeted to mitochondria (mito-GCaMP3). (A) Example images of mito-GCaMP3 at WT synapses before, during and after DLM motor axon stimulation (40 Hz for 5 sec) at 20 °C. Neuronal expression of the membrane-associated mCD8-mRFP protein provided a red fluorescent marker for nerve terminals. Times indicated are relative to the beginning of the time-lapse imaging and axon stimulation was initiated at 5 seconds. Note that mito-GCaMP3 exhibits a punctate distribution consistent with its mitochondrial localization (compare with the distribution of cytosolic GCaMP3 in Figure 9B). (B) Mito-GCaMP3 fluorescence changes (ΔF/F) elicited at WT and *dp32*^{EC1} (dp32EC1/Df) synapses by DLM motor axon stimulation (40 Hz for 5 seconds; bar over X axis) at 20 °C. Calcium transients observed in *dp32*^{EC1} closely resembled those of WT. Note that fluorescence intensity measurements were made in regions of interest corresponding to individual mito-GCaMP3 puncta (see panel A) and thus both the initial peak in DF/F as well as the sustained fluorescence increase following axon stimulation appear to reflect mitochondrial rather than cytosolic calcium changes.
