# Supplementary material for: Genetic Analysis in Drosophila Reveals a Role for the Mitochondrial Protein P32 in Synaptic Transmission
Source: G3 (Bethesda). 2012 Jan 1;2(1):59–69. doi: 10.1534/g3.111.001586 (PMC3276185; doi:10.1534/g3.111.001586)
Supplement: Supporting Information [file supp_2.1.59_FigureS1.pdf]

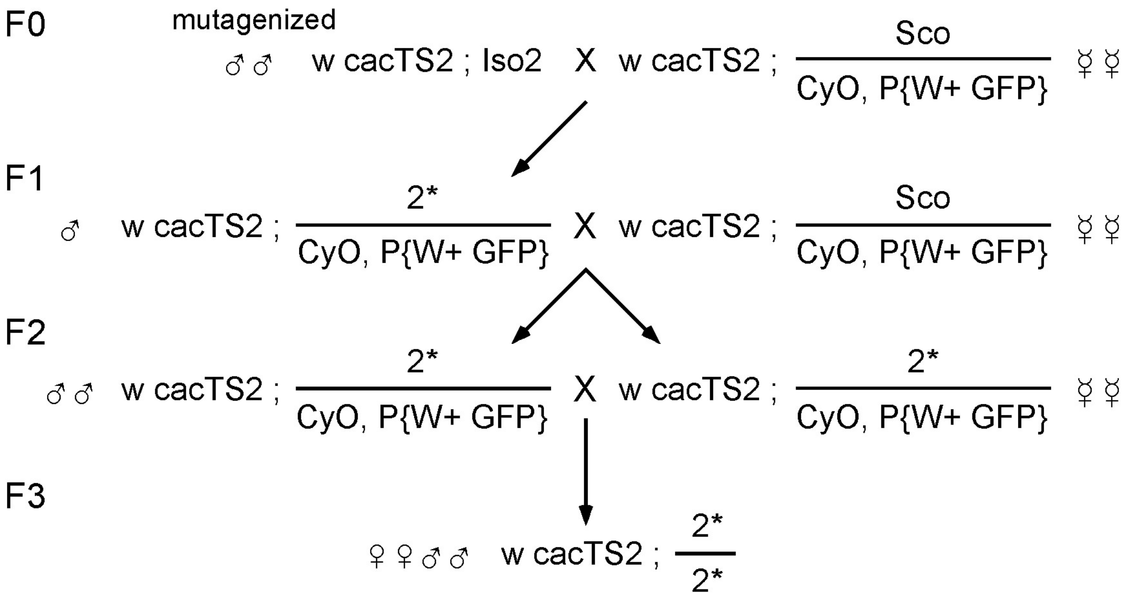

Figure S1 A Genetic Screen for Second Chromosome Modifiers of *cac*^{TS2}. *cac*^{TS2} males with an isogenized second chromosome were exposed to the mutagen, ethylmethane sulphonate (EMS). Mutagenized males were mated with *cac*^{TS2} females carrying the visible second chromosome marker, *Scutoid* (*Sco*), *in trans* to a *Curly of Oster* (*CyO*) balancer chromosome carrying a GFP transgene marked with w^+. The F1 male progeny were backcrossed to F0 females. After mating F2 heterozygous siblings, F3 flies homozygous for a mutagenized second chromosome in a *cac*^{TS2} genetic background were screened for altered *cac*^{TS2} behavior at 36°C. * ; mutagenized chromosome
