# Supplementary material for: Genetic Analysis in Drosophila Reveals a Role for the Mitochondrial Protein P32 in Synaptic Transmission
Source: G3 (Bethesda). 2012 Jan 1;2(1):59–69. doi: 10.1534/g3.111.001586 (PMC3276185; doi:10.1534/g3.111.001586)
Supplement: Supporting Information [file supp_2.1.59_FigureS2.pdf]

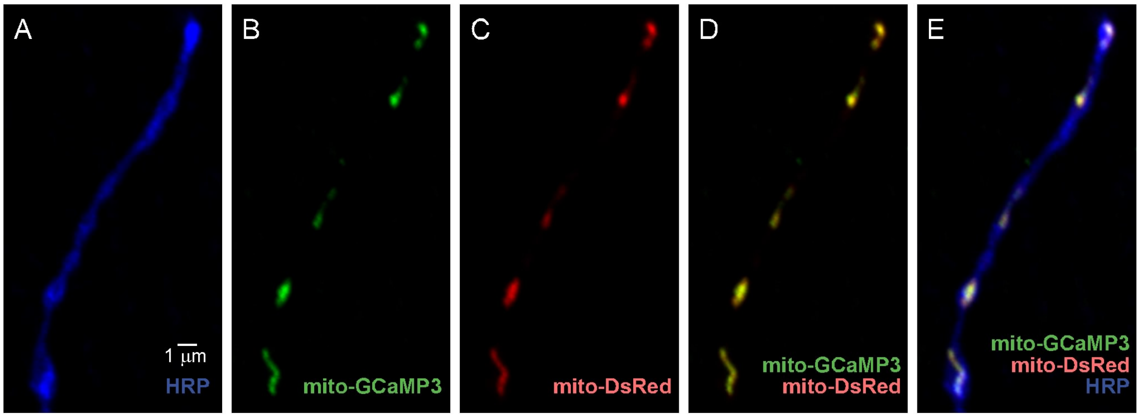

Figure S2 Localization of dP32 in Mitochondria at DLM Neuromuscular Synapses. Confocal immunofluorescence and native DsRed fluorescence images of adult DLM neuromuscular synapses exhibiting neuronal expression of mito-GCaMP3 and mito-DsRed. Mito-GCaMP3 consists of the first 71 amino acids of dP32 fused to the N-terminus of the calcium indicator, GCaMP3. Mito-GCaMP3 was recognized by an anti-GFP antibody. Mito-DsRed, which includes the previously characterized mitochondrial targeting domain of human COX8 (see Methods), serves as a mitochondrial marker. Anti-HRP labels the neuronal plasma membrane. Colocalization of mito-GCaMP3 and mito-DsRed demonstrates that mito-GCaMP3 was efficiently targeted to mitochondria.
