# Supplementary material for: Genetic Analysis in Drosophila Reveals a Role for the Mitochondrial Protein P32 in Synaptic Transmission
Source: G3 (Bethesda). 2012 Jan 1;2(1):59–69. doi: 10.1534/g3.111.001586 (PMC3276185; doi:10.1534/g3.111.001586)
Supplement: Supporting Information [file supp_2.1.59_FigureS3.pdf]

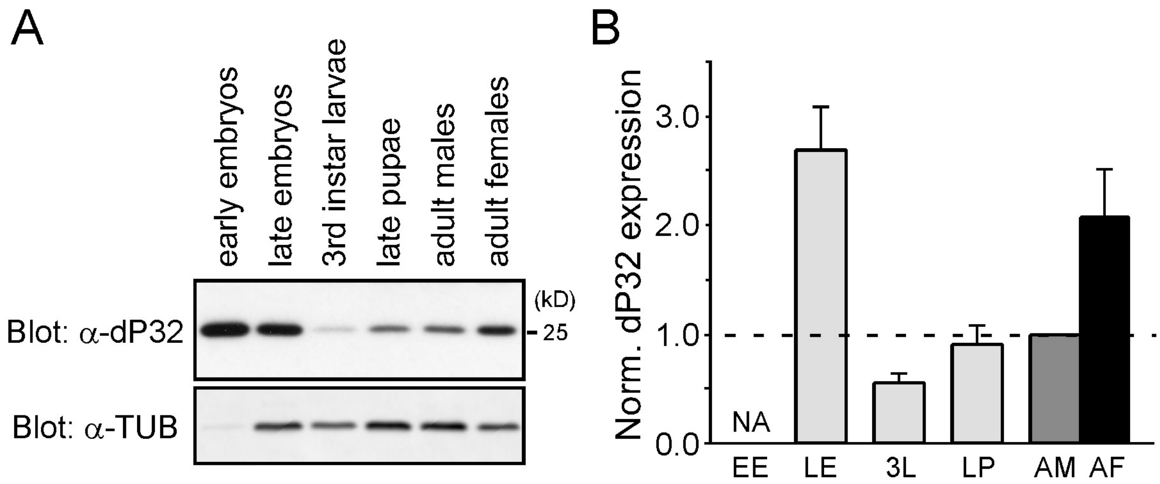

Figure S3 Developmental expression of dP32. Western analysis of whole animal homogenates prepared from WT at different stages of development: early embryos (EE), 0-4 hr after egg laying; late embryos (LE), 20-24 hr; third instar larvae (3L); late pupae (LP); adult males (AM) and females (AF). (A) Endogenous dP32 was recognized by an anti-dP32 antibody. Tubulin (TUB) was used as an internal loading control. (B) Comparison of average expression of dP32 at different stages of development obtained from three independent experiments. In each experiment, the dP32 signals at different stages were first divided by the corresponding Tubulin signal and the resulting values were normalized to those from adult male samples. The respective mean expression levels in LE, 3L, LP and AF were 2.68 ± 0.39, 0.56 ± 0.07, 0.91 ± 0.17 and 2.08 ± 0.44. NA (not applicable) indicates that quantitative analysis of the EE samples was not performed due to low expression of Tubulin at this stage.
