# Supplementary material for: Genetic Analysis in Drosophila Reveals a Role for the Mitochondrial Protein P32 in Synaptic Transmission
Source: G3 (Bethesda). 2012 Jan 1;2(1):59–69. doi: 10.1534/g3.111.001586 (PMC3276185; doi:10.1534/g3.111.001586)
Supplement: Supporting Information [file supp_2.1.59_FigureS4.pdf]

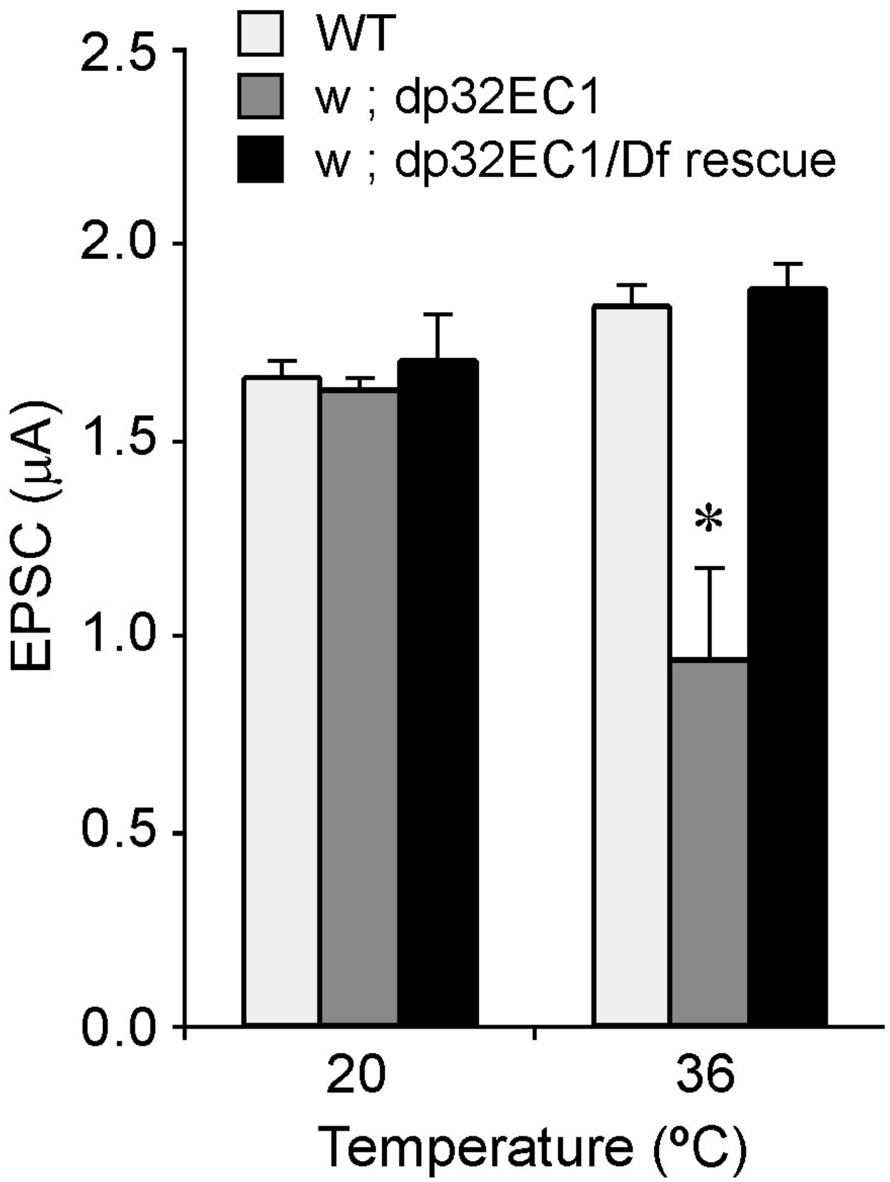

Figure S4 Presynaptic Expression of Wild-Type dP32 Rescues the *dp32*^{EC1} Synaptic Phenotype. Mean EPSC amplitudes in WT and the *dp32*^{EC1} mutant, as well as the *dp32*^{EC1} mutant expressing wild-type dP32 in the nervous system (dp32EC1/Df rescue). At 36°C, the EPSC amplitude in *dp32*^{EC1}*/Df* rescue [1.89 ± 0.07 µA (n = 5)] was not significantly different from that in WT (p = 0.65). The data for WT and *dp32*^{EC1}are the same as in Figure 2.
