# Supplementary material for: Genetic Analysis in Drosophila Reveals a Role for the Mitochondrial Protein P32 in Synaptic Transmission
Source: G3 (Bethesda). 2012 Jan 1;2(1):59–69. doi: 10.1534/g3.111.001586 (PMC3276185; doi:10.1534/g3.111.001586)
Supplement: Supporting Information [file supp_2.1.59_FigureS5.pdf]

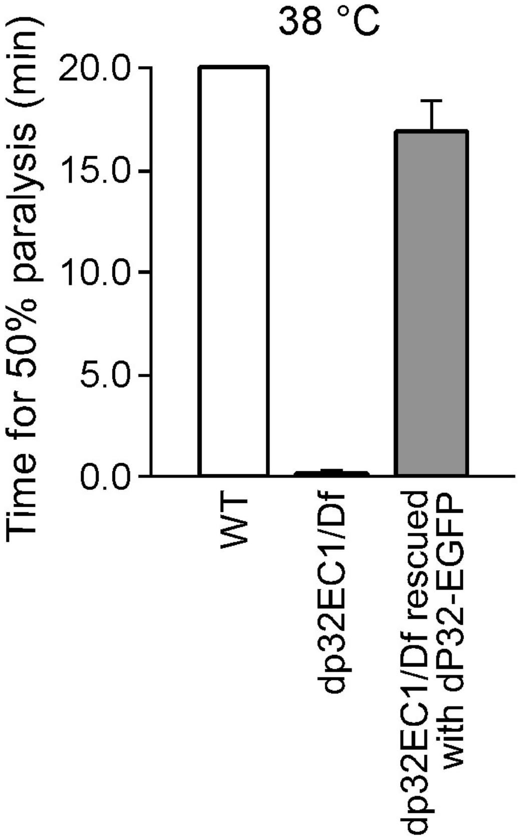

Figure S5 Presynaptic Expression of dP32-EGFP Rescues the *dp32*^{EC1} Paralytic Phenotype. Rescue of TS paralysis in the *dp32*^{EC1} mutant was examined at 38°C. The time for 50% paralysis was increased from 11.2 ± 3.12 sec (n = 5) in *dp32*^{EC1} (dp32EC1/Df) to 16.9 ± 1.48 min (n = 5) in *dp32*^{EC1} expressing dP32-EGFP in the nervous system (dp32EC1/Df rescued with dP32-EGFP). The WT behavioral test was truncated after 20 minutes. These results demonstrate that the dP32-EGFP fusion protein retains its function. The data for WT and *dp32*^{EC1}are the same as in Figure 1B.
