# Supplementary material for: Genetic Analysis in Drosophila Reveals a Role for the Mitochondrial Protein P32 in Synaptic Transmission
Source: G3 (Bethesda). 2012 Jan 1;2(1):59–69. doi: 10.1534/g3.111.001586 (PMC3276185; doi:10.1534/g3.111.001586)
Supplement: Supporting Information [file supp_2.1.59_FigureS6.pdf]

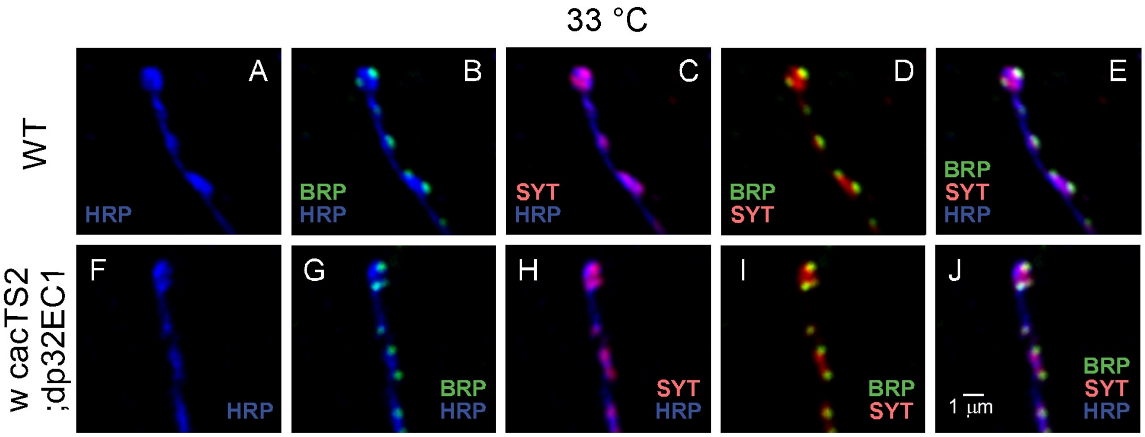

Figure S6 Preservation of Presynaptic Composition in the *dp32^{EC1}* Mutant. Confocal immunofluorescence images of adult DLM neuromuscular synapses in WT and *w cac^{TS2}; dp32^{EC1}* double mutants. Dissected preparations were exposed to a restrictive temperature of 33°C for 10 minutes and then fixed for processing. At this temperature, double mutant synapses exhibit a severe reduction in EPSC amplitude (Figure 2)]. Anti-HRP labels the neuronal plasma membrane such that the motor axon and its presynaptic boutons (swellings) are visualized. Anti-BRP labels presynaptic active zones. Anti-SYNAPTOTAGMIN (SYT) labels synaptic vesicles.
