# Supplementary material for: Genetic Analysis in Drosophila Reveals a Role for the Mitochondrial Protein P32 in Synaptic Transmission
Source: G3 (Bethesda). 2012 Jan 1;2(1):59–69. doi: 10.1534/g3.111.001586 (PMC3276185; doi:10.1534/g3.111.001586)
Supplement: Supporting Information [file supp_2.1.59_FigureS7.pdf]

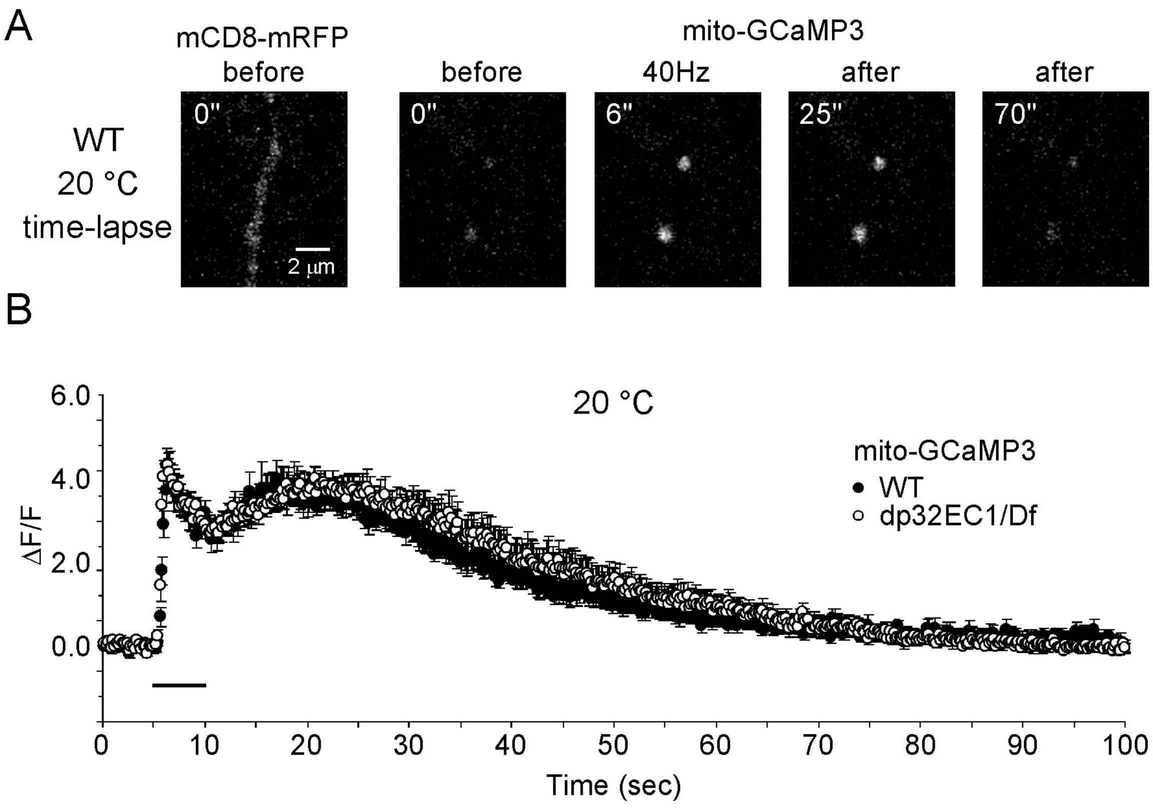

Figure S7 Mitochondrial Calcium Transients within DLM Neuromuscular Presynaptic Terminals. Imaging of mitochondrial calcium transients was performed at WT and *dp32*^{EC1} (dp32EC1/Df) synapses expressing the GCaMP3 targeted to mitochondria (mito-GCaMP3). (A) Example images of mito-GCaMP3 at WT synapses before, during and after DLM motor axon stimulation (40 Hz for 5 sec) at 20 °C. Neuronal expression of the membrane-associated mCD8-mRFP protein provided a red fluorescent marker for nerve terminals. Times indicated are relative to the beginning of the time-lapse imaging and axon stimulation was initiated at 5 seconds. Note that mito-GCaMP3 exhibits a punctate distribution consistent with its mitochondrial localization (compare with the distribution of cytosolic GCaMP3 in Figure 9B). (B) Mito-GCaMP3 fluorescence changes (ΔF/F) elicited at WT and *dp32*^{EC1} (dp32EC1/Df) synapses by DLM motor axon stimulation (40 Hz for 5 seconds; bar over X axis) at 20 °C. Calcium transients observed in *dp32*^{EC1} closely resembled those of WT. Note that fluorescence intensity measurements were made in regions of interest corresponding to individual mito-GCaMP3 puncta (see panel A) and thus both the initial peak in DF/F as well as the sustained fluorescence increase following axon stimulation appear to reflect mitochondrial rather than cytosolic calcium changes.
